# Crystallographic characterization of rare-earth cyano­tri­phenyl­borate complexes and the cyano­borates [NCBPh_3_]^1−^, [NCBPh_2_Me]^1−^, and [NCBPh_2_(μ-O)BPh_2_]^1−^

**DOI:** 10.1107/S2056989021006861

**Published:** 2021-07-13

**Authors:** Megan T. Dumas, Jessica R. K. White, Joseph W. Ziller, William J. Evans

**Affiliations:** a Department of Chemistry, University of California, Irvine, CA 92697-2025, USA

**Keywords:** lanthanide, rare earth, cyanide, cyano­tri­phenyl­borate, crystal structure

## Abstract

The investigation of the coordination chemistry of rare-earth metal complexes with cyanide ligands led to the isolation and crystallographic characterization of the *Ln* ^III^ cyano­tri­phenyl­borate complexes di­chlorido­(cyano­tri­phenyl­borato-κ*N*)tetra­kis­(tetra­hydro­furan-κ*O*)lanthanide(III), [*Ln*Cl_2_(C_19_H_15_BN)(C_4_H_8_O)_4_] [lanthanide (*Ln*) = dysprosium (Dy) and yttrium Y)] from reactions of LnCl_3_, KCN, and NaBPh_4_. Attempts to independently synthesize the tetra­ethyl­ammonium salt of (NCBPh_3_)^−^ from BPh_3_ and [NEt_4_][CN] in THF yielded crystals of the phenyl-substituted cyclic borate, tetra­ethyl­aza­nium 2,2,4,6-tetra­phenyl-1,3,5,2λ^4^,4,6-trioxatriborinan-2-ide, C_8_H_20_N^+^·C_24_H_20_B_3_O_3_^−^ or [NEt_4_][B_3_(μ-O)_3_(C_6_H_5_)_4_]. The mechanochemical reaction of BPh_3_ and [NEt_4_][CN] without solvent produced crystals of tetra­ethyl­aza­nium cyano­diphenyl-λ^4^-boranyl di­phenyl­borinate, C_8_H_20_N^+^·C_25_H_20_B_2_NO^−^ or [NEt_4_][NCBPh_2_(μ-O)BPh_2_]. Reaction of BPh_3_ and KCN in THF in the presence of 2.2.2-cryptand (crypt) led to a crystal of bis­[(2.2.2-cryptand)potassium] 2,2,4,6-tetra­phenyl-1,3,5,2λ^4^,4,6-trioxatriborinan-2-ide cyano­methyl­diphenyl­borate tetra­hydro­furan disolvate, 2C_18_H_36_KN_2_O_6_^+^·C_24_H_20_B_3_O_3_^−^·C_14_H_13_BN^−^·2C_4_H_8_O or [K(crypt)]_2_[B_3_(μ-O)_3_(C_6_H_5_)_4_][NCBPh_2_Me]·2THF. The [NCBPh_2_(μ-O)BPh_2_]^1−^ and (NCBPh_2_Me)^1−^ anions have not been structurally characterized previously. The structure of **1-Y** was refined as a two-component twin with occupancy factors 0.513 (1) and 0.487 (1). In **4**, one solvent mol­ecule was disordered and included using multiple components with partial site-occupancy factors.

## Abstract 2

The investigation of the coordination chemistry of rare-earth metal complexes with cyanide ligands led to the isolation and crystallographic characterization of the *Ln* ^III^ cyano­tri­phenyl­borate complexes, *Ln*Cl_2_(THF)_4_(NCBPh_3_) (*Ln* = Dy, Y) as well as the cyano­borates [NEt_4_][B_3_(μ-O)_3_(C_6_H_5_)_4_], [NEt_4_][NCBPh_2_(μ-O)BPh_2_], [K(crypt)]_2_[B_3_(μ-O)_3_(C_6_H_5_)_4_][NCBPh_2_Me]. The [NCBPh_2_(μ-O)BPh_2_]^1−^ and (NCBPh_2_Me)^1−^ anions have not been structurally characterized previously.

## Chemical context   

Attempts to make cationic rare-earth metal cyanide complexes of the type [*Ln*(CN)_2_(THF)_x_][BPh_4_] by combining *Ln*Cl_3_ with sodium tetra­phenyl­borate and potassium cyanide led to the isolation of the cyano­tri­phenyl­borate complexes *Ln*Cl_2_(THF)_4_(NCBPh_3_), **1-** ***Ln*** (*Ln* = Dy, Y). Previously, transition-metal complexes of (NCBPh_3_)^1−^ have been known to form from RhCl(PPh_3_)_3_, KCN, and BPh_3_ (Pankowski *et al.*, 1996 ▸; Carlton *et al.*, 1998 ▸; Fernandes *et al.*, 2002 ▸) and from [Et_4_N][Cr(CN)_6_] and BPh_3_ (Schelter *et al.*, 2005 ▸).

Efforts to independently synthesize the tetra­ethyl­ammonium salt of the (NCBPh_3_)^1−^ ligand generated a borate anion and two new cyano­phenyl­borate anions that, to our knowledge, have not been structurally characterized. Specifically, the reaction of BPh_3_ and [NEt_4_][CN] in THF led to crystals of the cyclic borate, [NEt_4_][B_3_(μ-O)_3_(C_6_H_5_)_4_], **2**. When the analogous reaction was tried mechanochemically without solvent, the cyano­borate, [NEt_4_][NCBPh_2_(μ-O)BPh_2_], **3**, was obtained. Reaction of BPh_3_ with KCN in the presence of 2.2.2-cryptand (crypt) gave crystals of the double salt [K(crypt)]_2_[B_3_(μ-O)_3_Ph_4_][NCBPh_2_Me], **4**. The cyano­borate anions in **3** and **4** have not been previously characterized by X-ray crystallography. The *ChemDraw* representations of **1-** ***Ln*** (*Ln* = Dy, Y), **2**, **3**, and **4** are depicted in the scheme below. 
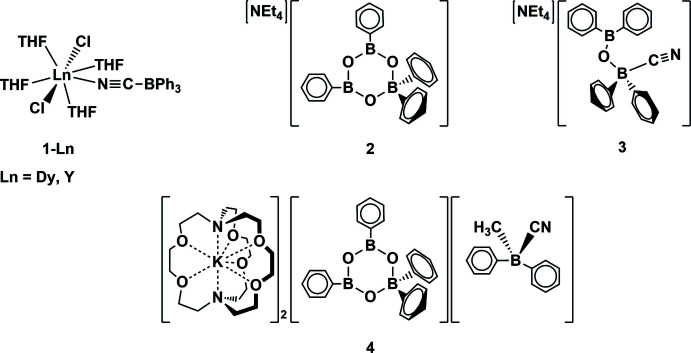


## Structural commentary   

**Figure 1:**
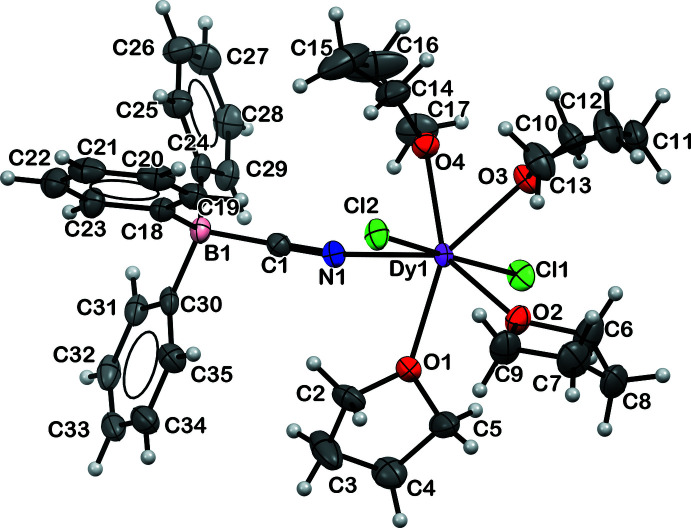
Displacement ellipsoid plot of DyCl_2_(THF)_4_(NCBPh_3_), **1-Dy**, drawn at the 30% probability level.

The displacement ellipsoid plot of *Ln*Cl_2_(THF)_4_(NCBPh_3_) **1-** ***Ln*** (*Ln* = Dy, Y) is depicted in Fig. 1 ▸ and the structural parameters are tabulated in Table 1 ▸. **1-Dy** and **1-Y** crystallize in the *P* 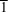 space group and are isomorphous. The geometry around the Ln^III^ ions is distorted penta­gonal bipyramidal. The C1—N1 bond distances are 1.141 (3) and 1.144 (4) Å in **1-Dy** and **1-Y**, respectively. These distances are consistent with a C≡N triple bond (Allen *et al.*, 1987 ▸). The 178.7 (3) and 178.4 (3)° N1—C1—B1 bond angles in **1-Dy** and **1-Y**, respectively, are also consistent with a C≡N triple bond. The C1—N1—*Ln*1 angles are 163.92 (19) and 164.6 (3)° in **1-Dy** and **1-Y**, respectively. In comparison, the titanium complex, [(*η* ^5^-C_5_H_5_)_2_Ti(*η* ^2^-COR)(NCBPh_3_)], has a C—N distance of 1.14 (2) Å, an N—C—B angle of 176.8 (16)°, and a C—N—Ti angle of 169.1 (13)° (Pankowski *et al.*, 1996 ▸). The chromium complex [Et_4_N]_3_[Cr(NCBPh_3_)_6_] has C—N distances of 1.127 (5), 1.100 (5), and 1.150 (5) Å, N—C—B angles of 178.9 (4), 176.8 (4), and 179.8 (5)°, and Cr—N—C angles of 174.8 (3), 175.4 (3), and 173.5 (3)° (Schelter *et al.*, 2005 ▸). The B—C(CN) and B—C(phen­yl) distances in **1-Dy** and **1-Y**, respectively, are similar to those reported in transition-metal complexes with [NCBPh_3_]^1−^ ligands (Pankowski *et al.*, 1996 ▸; Fernandes *et al.*, 2002 ▸).

**Figure 2:**
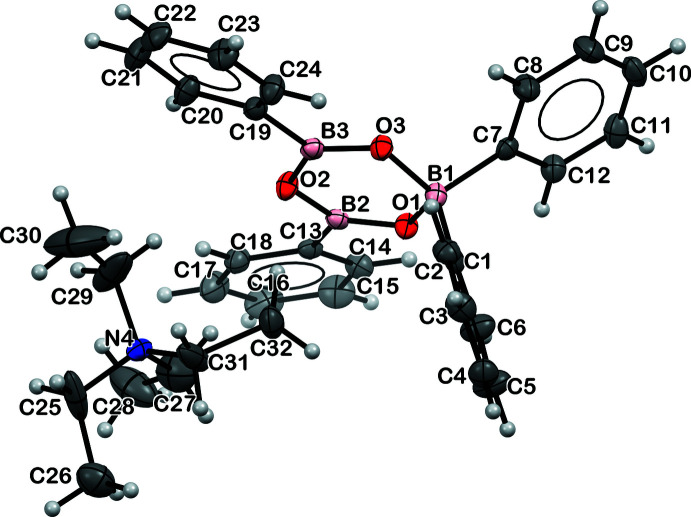
Displacement ellipsoid plot of **2**, [NEt_4_][B_3_(μ-O)_3_(C_6_H_5_)_4_], drawn at the 30% probability level. The disorder in the [NEt_4_]^+^ cation is omitted for clarity.

The displacement ellipsoid plots of [NEt_4_][B_3_(μ-O)_3_(C_6_H_5_)_4_], **2**, [NEt_4_][NCBPh_2_(μ-O)BPh_2_], **3**, and [K(crypt)]_2_[B_3_(μ-O)_3_(C_6_H_5_)_4_][NCBPh_2_Me], **4**, are shown below in Figs. 2 ▸, 3 ▸ and 4 ▸, respectively, and the structural parameters are tabulated in Table 1 ▸. Both **2** and **4** contain a non-planar anionic tetra­phenyl­boroxinate ring as reported previously in the tetra­methyl­ammonium salt, [NMe_4_][B_3_(μ-O)_3_(C_6_H_5_)_4_] (Kliegel *et al.*, 1986 ▸). While complex **2** is a tetra­ethyl­ammonium salt of the tetra­phenyl­boroxinate anion, complex **4** is a double salt with the second anion being [NCBPh_2_Me]^1−^. There are no crystallographically characterized examples of this anion in the literature to our knowledge. Complex **3** contains a [Ph_2_B(μ-O)BPh_2_CN]^1−^ anion, differing both from the cyclic B_3_O_3_ phenyl­boroxinate anions found in **2** and **4** and from the [NCBPh_2_Me]^1−^ anion in **4**. We found no crystallographically characterized examples of this anion in the literature.

The O—B—O and B—O—B angles range from 110.4 (1) to 122.5 (2)° for **2** and range from 109.9 (5) to 123.3 (5)° for **4**, both ranges are similar to those in [NMe_4_][B_3_(μ-O)_3_(C_6_H_5_)_4_]. The 1.497 (2) and 1.504 (2) Å B—O bonds involving four-coordinate B1 in **2** are longer than the other four B—O bond distances involving three-coordinate boron, which range from 1.333 (2) Å to 1.399 (2) Å for **2**. Similar distances are found in **4**.

Inter­estingly, though the B–C(CN) and B–C(phen­yl) distances for **4** are similar to those of the [NCBPh_3_]^−^ anion in **1-** ***Ln***, the N—C—B angle of 171.6 (9)° in **4** is less linear than the N—C—B angles in **1-** ***Ln*** and in **3**, 178 and 177.10 (13)°, respectively.

Both the C—N and B—C(CN) lengths in **3** and **4** are similar to those in **1-** ***Ln***. The 1.488 (10) Å B4—C(Me) distance in **4** is much shorter than the reported B—C(Me) distances in [BMe_4_]^−^ [1.639 (2) to 1.648 (2) Å] (Zhu & Kochi, 1999 ▸), [BPh_3_Me]^−^ [1.653 (2) Å] (Zhu & Kochi, 1999 ▸), and [FcB(Mes^F^)(Me)(CN)]^−^ [1.628 (5) Å] (Broomsgrove *et al.*, 2010 ▸). The B4—C(Me) distance is also shorter than the 1.636 (2) and 1.614 (10) Å B—C(CN) distances in **3** and **4**, respectively. The 1.633 (11) and 1.648 (11) Å B4—C(phen­yl) distances are similar to the other B—C(phen­yl) distances in the structures reported here.

## Supra­molecular features   

There are no notable supra­molecular features in **1-** ***Ln***, **2**, **3**, or **4**.

## Database survey   

The Cambridge Structural Database contains 14 known structures of transition metal complexes with the [NCBPh_3_]^1−^ ligand found in **1-** ***Ln*** or related [NCB(C_6_F_5_)_3_]^1−^ ligands, including titanium (Pankowski *et al.*, 1996 ▸, CSD refcode: TEXLEN), chromium (Schelter *et al.*, 2005 ▸, CSD Refcode: XAKCUI), iron (Vei *et al.*, 2003 ▸, CSD Refcode: TAGKUI), nickel (Brunkan *et al.*, 2004 ▸, CSD Refcode: AVOKAX), copper (Naza­renko *et al.*, 1996 ▸, CSD Refcode: REYHEI) zirconium (Zhou *et al.*, 2001 ▸, CSD Refcode: YEQZEZ), and rhodium (Fernandes *et al.*, 2002 ▸, CSD Refcode: XUTDIZ; Cornock *et al.*, 1977 ▸, CSD Refcode: CBORRH). There is also a crystallographically-characterized cerium cyano­tri­phenyl­borate complex, [Ce(L_OEt_)_2_(NCBPh_3_)_2_] (L_OEt_^−^ = [Co(*η* ^5^-C_5_H_5_){P(O)(OEt)_2_}_3_]^−^) (Au-Yeung *et al.*, 2016 ▸, CSD Refcode: EYAZOV).

Other crystallographically characterized tetra­phenyl­boroxinates of the type found in **2** that are reported in the Cambridge Structural Database include [Me_3_NCH_2_CH_2_OH][B_3_(μ-O)_3_(C_6_H_5_)_4_] (Beckett *et al.*, 2006 ▸, CSD Refcode: ICUWAF), [NEt_3_H][B_3_(μ-O)_3_(C_6_H_5_)_4_] (Kratzert & Krossing, 2018 ▸, CSD Refcode: HERJUM01), [(^*t*^Bu_3_PAu)_4_P][B_3_(μ-O)_3_(C_6_H_5_)_4_] (Zeller *et al.*, 1993 ▸, CSD Refcode: PEVZOF), [C_6_H_11_NMe_3_][B_3_(μ-O)_3_(C_6_H_5_)_4_] (Beckett *et al.*, 2018 ▸, CSD Refcode: VEKVIT), [Ph_2_B{OCH_2_CH_2_N(Me)(CH_2_)_*n*_}_2_][B_3_(μ-O)_3_(C_6_H_5_)_4_] (*n* = 4, 5) (Beckett *et al.*, 2010 ▸, CSD Refcode: VUTGUN), and [(*η^5^*-C_5_H_5_)Ni(*η^6^*-C_6_H_6_)Ni(*η^5^*-C_5_H_5_)][B_3_(μ-O)_3_(C_6_F_5_)_5_] (Priego *et al.*, 2000 ▸, CSD Refcode: MEKLAP).

There are no crystallographically characterized examples of the [Ph_2_B(μ-O)BPh_2_CN]^1−^ and [NCBPh_2_Me]^1−^ anions found in **3** and **4**, respectively, in the literature.

## Synthesis and crystallization   

**DyCl_2_(THF)_4_(NCBPh_3_)**, **1-Dy.** In an argon-filled glovebox, KCN (42 mg, 0.642 mmol) was added to a stirred slurry of DyCl_3_ (75 mg, 0.279 mmol) in THF (10 mL). NaBPh_4_ (96 mg, 0.279 mmol) was added to the stirred slurry. The cloudy white solution was stirred overnight. The volatiles were removed under vacuum. The product was extracted into THF (10 mL) and centrifuged to remove white solids. The clear colorless solution had its volatiles removed under vacuum. The product was isolated as a colorless powder. Colorless crystals of DyCl_2_(THF)_4_(NCBPh_3_), **1-Dy**, suitable for X-ray diffraction, were isolated from a vapor diffusion of hexane into a concentrated THF solution at room temperature after 6 d.

**YCl_2_(THF)_4_(NCBPh_3_), 1-Y.** In an argon-filled glovebox, KCN (34 mg, 0.522 mmol) was stirred in THF (10 mL) to form a cloudy white slurry. After 4 h, YCl_3_ (51 mg, 0.260 mmol) was tapped into the stirred white slurry. After 5 min, a solution of NaBPh_4_ (89 mg, 0.260 mmol) in THF (8 mL) was added to the stirred slurry. The cloudy white slurry was stirred overnight. The white slurry was centrifuged. The clear, colorless supernatant was collected, and the centrifuge pellet was washed with THF (5 mL), and the wash was combined with the supernatant. The colorless solution had its volatiles removed under vacuum. The product was isolated as a colorless solid (107 mg). X-ray quality crystals were isolated from a vapor diffusion of hexane into a concentrated THF solution at room temperature after 10 d.

**[NEt_4_][B_3_(** ***μ*** **-O)_3_(C_6_H_5_)_4_], 2.** In an argon-filled glovebox, BPh_3_ (78 mg, 0.320 mmol) was tapped into a stirred slurry of Et_4_NCN (50 mg, 0.320 mmol) in THF (5 ml). THF (10 mL) was added to the slurry, and the solution was heated lightly on a hot plate to encourage the Et_4_NCN to dissolve. After the sample was heated lightly for about an hour, most of the white solids had dissolved. The solution was allowed to stir at room temperature overnight. The volatiles were removed under vacuum and the white solids were washed with toluene (2 mL) twice. The thick tacky colorless solids were extracted into THF. Colorless X-ray quality crystals of [NEt_4_][B_3_(μ-O)_3_(C_6_H_5_)_4_], **2**, were grown from a slow evaporation of a THF solution at room temperature.

**[NEt_4_][NCBPh_2_(** ***μ*** **-O)BPh_2_], 3.** In an argon-filled glovebox, BPh_3_ and NEt_4_CN were added to a BMT-20-S tube drive along with 40 steel balls (6 mm). The reaction mixture was ball milled together for 40 minutes using an Ultra-Turrax Tube Drive at the maximum speed setting. After this time, the colorless solids were extracted into toluene and THF. The volatiles were removed under vacuum. X-ray quality colorless crystals of [NEt_4_][NCBPh_2_(μ-O)BPh_2_], **3**, were grown from a slow evaporation of a concentrated THF solution at 258 K after a few days.

**[K(crypt)]_2_[B_3_(** ***μ*** **-O)_3_(C_6_H_5_)_4_][NCBPh_2_Me]**, **4**. In an argon-filled glovebox, 2.2.2-cryptand (156 mg, 0.413 mmol) and KCN (30 mg, 0.460 mmol) were stirred in THF (10 mL) for 2 h, which allowed most of the white solids to dissolve. A solution of BPh_3_ (100 mg, 0.413 mmol) in THF (5 mL) was added to this mixture and the combination was allowed to stir for 3 d. The volatiles were removed under vacuum from the clear and colorless solution. The sample was extracted into THF (20 mL) and volatiles were removed under vacuum. The product was isolated as a colorless solid (423 mg). Colorless X-ray quality crystals of [K(crypt)]_2_[B_3_(μ-O)_3_(C_6_H_5_)_4_][NCBPh_2_Me], **4**, were obtained from a vapor diffusion of pentane into a concentrated THF solution at room temperature.

## Refinement   

**General Structure Solution and Refinement.** The analytical scattering factors (Wilson, 1992 ▸) for neutral atoms were used throughout the analysis. Hydrogen atoms were included using a riding model. **DyCl_2_(THF)_4_(NCBPh_3_), 1-Dy**: Data were collected using a 15 sec/frame scan time. There were no systematic absences nor any diffraction symmetry other than the Friedel condition. Atom C5 was disordered and included using multiple components with partial site-occupancy factors. **YCl_2_(THF)_4_(NCBPh_3_), 1-Y**: Data were collected using a 30 sec/frame scan time. There were no systematic absences nor any diffraction symmetry other than the Friedel condition. Disordered atoms were included using multiple components with partial site-occupancy-factors. The structure was refined as a two-component twin with occupancy factors 0.513 (1) and 0.487 (1). **[NEt_4_][B_3_(** ***μ*** **-O)_3_(C_6_H_5_)_4_], 2**: Data were collected using a 20 sec/frame scan time. The tetra­ethyl­ammonium ion was fully disordered. The disordered atoms were included using multiple components with partial site-occupancy factors. **[NEt_4_][NCBPh_2_(** ***μ*** **-O)BPh_2_], 3**: Data were collected using a 30 sec/frame scan time. **[K(crypt)]_2_[B_3_(** ***μ*** **-O)_3_(C_6_H_5_)_4_][NCBPh_2_Me], 4**: Data were collected using a 60 sec/frame scan time. There were two mol­ecules of tetra­hydro­furan solvent present. One solvent mol­ecule was disordered and included using multiple components with partial site-occupancy factors. Crystal data, data collection and structure refinement details are summarized in Table 2 ▸.

## Supplementary Material

Crystal structure: contains datablock(s) 1-Dy, 1-Y, 2, 3, 4. DOI: 10.1107/S2056989021006861/pk2658sup1.cif

Structure factors: contains datablock(s) 1-Dy. DOI: 10.1107/S2056989021006861/pk26581-Dysup7.hkl

Structure factors: contains datablock(s) 1-Y. DOI: 10.1107/S2056989021006861/pk26581-Ysup8.hkl

Structure factors: contains datablock(s) 2. DOI: 10.1107/S2056989021006861/pk26582sup4.hkl

Structure factors: contains datablock(s) 3. DOI: 10.1107/S2056989021006861/pk26583sup5.hkl

Structure factors: contains datablock(s) 4. DOI: 10.1107/S2056989021006861/pk26584sup6.hkl

CCDC references: 2094137, 2094136, 2094135, 2094134, 2094133

Additional supporting information:  crystallographic information; 3D view; checkCIF report

## Figures and Tables

**Figure 3:**
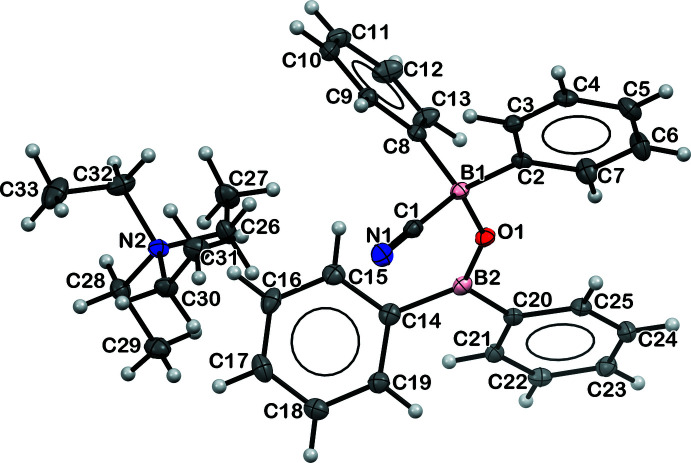
Displacement ellipsoid plot of **3**, [NEt_4_][NCBPh_2_(μ-O)BPh_2_], drawn at the 50% probability level.

**Figure 4:**
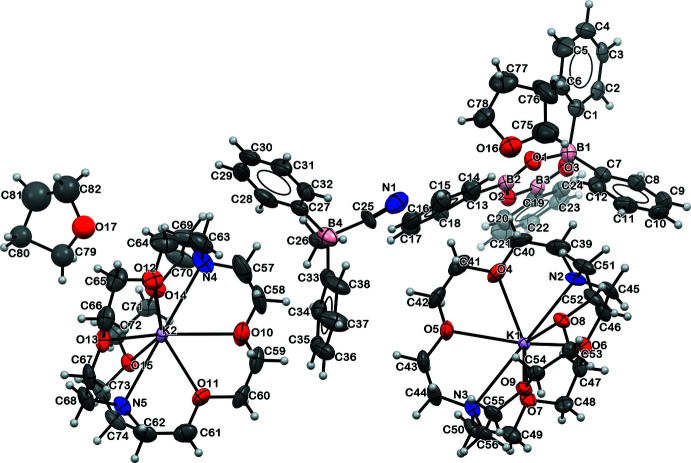
Displacement ellipsoid plot of **4**, [K(crypt)]_2_[B_3_(μ-O)_3_(C_6_H_5_)_4_][NCBPh_2_Me], drawn at the 50% probability level.

**Table 1:** Selected bond lengths and angles (Å, °) for **1-Dy** and **1-Y**

	**1-Dy**	**1-Y**
*Ln*1—O1	2.3790 (18)	2.369 (2)
*Ln*1—O2	2.3838 (17)	2.370 (2)
*Ln*1—O3	2.4022 (16)	2.390 (2)
*Ln*1—O4	2.3932 (17)	2.382 (2)
*Ln*1—N1	2.431 (2)	2.420 (3)
*Ln*1—Cl1	2.5888 (6)	2.5803 (9)
*Ln*1—Cl2	2.5835 (6)	2.5730 (8)
N1—C1	1.141 (3)	1.144 (4)
B1—C1	1.621 (3)	1.629 (4)
B1—C18	1.625 (3)	1.630 (5)
B1—C24	1.632 (4)	1.626 (5)
B1—C30	1.641 (3)	1.642 (5)
		
C1—N1—*Ln*1	163.92 (19)	164.6 (3)
N1—C1—B1	178.7 (3)	178.4 (3)

**Table 2:** Experimental details

	**1-Dy**	**1-Y**	**2**	**3**	**4**
Crystal data					
Chemical formula	[DyCl_2_(C_19_H_15_BN)(C_4_H_8_O)_4_]	[YCl_2_(C_19_H_15_BN)(C_4_H_8_O)_4_]	C_8_H_20_N^+^·C_24_H_20_B_3_O_3_^−^	C_8_H_20_N^+^·C_25_H_20_B_2_NO^−^	2C_18_H_36_KN_2_O_6_^+^·C_24_H_20_B_3_O_3_^−^·C_14_H_13_BN^−^·2C_4_H_8_O
*M*_r_	789.94	716.35	519.08	502.29	1570.27
Crystal system, space group	Triclinic, *P*\overline{1}	Triclinic, *P*\overline{1}	Monoclinic, *P*2_1_/*n*	Monoclinic, *P*2_1_/*n*	Orthorhombic, *P* *c* *a*2_1_
Temperature (K)	158	173	133	88	88
*a*, *b*, *c* (Å)	12.0043 (17), 12.5261 (17), 13.4913 (19)	12.0404 (9), 12.5428 (9), 13.4654 (10)	9.7245 (7), 18.3021 (13), 16.7716 (12)	11.0269 (8), 13.9387 (11), 18.8488 (14)	27.193 (2), 14.5520 (11), 21.2218 (16)
α, β, γ (°)	114.7161 (13), 101.2230 (16), 94.2256 (16)	114.6967 (8), 101.3142 (9), 94.3882 (9)	90, 101.5456 (9), 90	90, 100.6357 (10), 90	90, 90, 90
*V* (Å^3^)	1780.0 (4)	1782.7 (2)	2924.6 (4)	2847.3 (4)	8397.7 (11)
*Z*	2	2	4	4	4
Radiation type	Mo *K*α	Mo *K*α	Mo *K*α	Mo *K*α	Mo *K*α
μ (mm^−1^)	2.29	1.82	0.07	0.07	0.18
Crystal size (mm)	0.39 × 0.33 × 0.26	0.32 × 0.20 × 0.13	0.37 × 0.33 × 0.23	0.28 × 0.27 × 0.22	0.45 × 0.33 × 0.28

Data collection					
Diffractometer	Bruker SMART APEXII CCD	Bruker SMART APEXII CCD	Bruker SMART APEXII CCD	Bruker SMART APEXII CCD	Bruker SMART APEXII CCD
Absorption correction	Numerical (*SADABS*; Krause *et al.*, 2015 ▸)	Multi-scan (*TWINABS*; Sheldrick, 2012 ▸)	Multi-scan (*SADABS*; Krause *et al.*, 2015 ▸)	Multi-scan (*SADABS*; Krause *et al.*, 2015 ▸)	Multi-scan (*SADABS*; Krause *et al.*, 2015 ▸)
*T*_min_, *T*_max_	0.558, 0.696	0.576, 0.746	0.834, 0.862	0.715, 0.746	0.825, 0.862
No. of measured, independent and observed [*I* > 2σ(*I*)] reflections	21440, 8460, 7562	7988, 7988, 6188	31741, 5959, 5030	34768, 7242, 5500	85621, 15958, 14236
*R*_int_	0.019	–	0.029	0.042	0.038
(sin θ/λ)_max_ (Å^−1^)	0.680	0.650	0.625	0.685	0.610

Refinement					
*R*[*F* ^2^ > 2σ(*F* ^2^)], *wR*(*F* ^2^), *S*	0.024, 0.061, 1.04	0.047, 0.102, 1.04	0.064, 0.179, 1.02	0.048, 0.119, 1.04	0.074, 0.209, 1.06
No. of reflections	8460	7988	5959	7242	15958
No. of parameters	406	425	442	347	988
No. of restraints	0	0	0	0	1
H-atom treatment	H-atom parameters constrained	H-atom parameters constrained	H-atom parameters constrained	H-atom parameters constrained	H-atom parameters constrained
Δρ_max_, Δρ_min_ (e Å^−3^)	1.34, −0.89	0.64, −0.81	1.06, −0.47	0.41, −0.24	0.78, −0.34

Computer programs: *APEX2* (Bruker, 2014 ▸), *SAINT* (Bruker, 2013 ▸), *SHELXT2014/4* (Sheldrick, 2015*a* ▸), *SHELXL2014/7* (Sheldrick, 2015*b* ▸), and *SHELXTL* (Sheldrick, 2008 ▸).

## supplementary crystallographic information

### Dichlorido(cyanotriphenylborato-κN)tetrakis(tetrahydrofuran-κO)dysprosium(III) (1-Dy) Crystal data

**Table:**

[DyCl_2_(C_19_H_15_BN)(C_4_H_8_O)_4_]	*Z* = 2
*M**_r_* = 789.94	*F*(000) = 802
Triclinic, *P*1	*D*_x_ = 1.474 Mg m^−^^3^
*a* = 12.0043 (17) Å	Mo *K*α radiation, λ = 0.71073 Å
*b* = 12.5261 (17) Å	Cell parameters from 9908 reflections
*c* = 13.4913 (19) Å	θ = 2.3–28.8°
α = 114.7161 (13)°	µ = 2.29 mm^−^^1^
β = 101.2230 (16)°	*T* = 158 K
γ = 94.2256 (16)°	Irregular, colorless
*V* = 1780.0 (4) Å^3^	0.39 × 0.33 × 0.26 mm

### Dichlorido(cyanotriphenylborato-κN)tetrakis(tetrahydrofuran-κO)dysprosium(III) (1-Dy) Data collection

**Table:**

Bruker SMART APEXII CCD diffractometer	7562 reflections with *I* > 2σ(*I*)
Radiation source: fine-focus sealed tube	*R*_int_ = 0.019
φ and ω scans	θ_max_ = 28.9°, θ_min_ = 1.7°
Absorption correction: numerical (SADABS; Krause et al., 2015)	*h* = −16→16
*T*_min_ = 0.558, *T*_max_ = 0.696	*k* = −16→16
21440 measured reflections	*l* = −18→18
8460 independent reflections	

### Dichlorido(cyanotriphenylborato-κN)tetrakis(tetrahydrofuran-κO)dysprosium(III) (1-Dy) Refinement

**Table:**

Refinement on *F*^2^	Primary atom site location: dual space
Least-squares matrix: full	Secondary atom site location: difference Fourier map
*R*[*F*^2^ > 2σ(*F*^2^)] = 0.024	Hydrogen site location: inferred from neighbouring sites
*wR*(*F*^2^) = 0.061	H-atom parameters constrained
*S* = 1.04	*w* = 1/[σ^2^(*F*_o_^2^) + (0.0251*P*)^2^ + 2.2673*P*] where *P* = (*F*_o_^2^ + 2*F*_c_^2^)/3
8460 reflections	(Δ/σ)_max_ = 0.002
406 parameters	Δρ_max_ = 1.34 e Å^−^^3^
0 restraints	Δρ_min_ = −0.89 e Å^−^^3^

### Dichlorido(cyanotriphenylborato-κN)tetrakis(tetrahydrofuran-κO)dysprosium(III) (1-Dy) Special details

**Table:**

Geometry. All esds (except the esd in the dihedral angle between two l.s. planes) are estimated using the full covariance matrix. The cell esds are taken into account individually in the estimation of esds in distances, angles and torsion angles; correlations between esds in cell parameters are only used when they are defined by crystal symmetry. An approximate (isotropic) treatment of cell esds is used for estimating esds involving l.s. planes.
Refinement. A colorless crystal of approximate dimensions 0.255 x 0.332 x 0.391 mm was mounted in a cryoloop and transferred to a Bruker SMART APEX II diffractometer. The APEX2 program package was used to determine the unit-cell parameters and for data collection (15 sec/frame scan time for a sphere of diffraction data). The raw frame data was processed using SAINT and SADABS to yield the reflection data file. Subsequent calculations were carried out using the SHELXTL program. There were no systematic absences nor any diffraction symmetry other than the Friedel condition. The centrosymmetric triclinic space group P-1 was assigned and later determined to be correct.The structure was solved by dual space methods and refined on F2 by full-matrix least-squares techniques. The analytical scattering factors for neutral atoms were used throughout the analysis. Hydrogen atoms were included using a riding model. Atom C(5) was disordered and included using multiple components with partial site-occupancy-factors.Least-squares analysis yielded wR2 = 0.0609 and Goof = 1.042 for 406 variables refined against 8460 data (0.74Å), R1 = 0.0239 for those 7562 data with I > 2.0sigma(I).

### Dichlorido(cyanotriphenylborato-κN)tetrakis(tetrahydrofuran-κO)dysprosium(III) (1-Dy) Fractional atomic coordinates and isotropic or equivalent isotropic displacement parameters (Å^2^)

**Table:**

	*x*	*y*	*z*	*U*_iso_*/*U*_eq_	Occ. (<1)
Dy1	0.24691 (2)	0.24994 (2)	0.49865 (2)	0.01889 (4)	
Cl1	0.23261 (5)	0.07841 (6)	0.55751 (5)	0.03227 (13)	
Cl2	0.25400 (5)	0.40466 (5)	0.41894 (5)	0.03005 (12)	
O1	0.45097 (15)	0.26894 (16)	0.55203 (16)	0.0342 (4)	
O2	0.30435 (16)	0.12131 (15)	0.33601 (14)	0.0316 (4)	
O3	0.07375 (14)	0.14589 (15)	0.35225 (14)	0.0287 (4)	
O4	0.08323 (15)	0.30171 (15)	0.57396 (14)	0.0288 (4)	
N1	0.31345 (17)	0.42077 (18)	0.68261 (17)	0.0284 (4)	
B1	0.3578 (2)	0.6482 (2)	0.8573 (2)	0.0236 (5)	
C1	0.33280 (19)	0.5144 (2)	0.75535 (19)	0.0247 (5)	
C2	0.5339 (2)	0.3715 (3)	0.5732 (3)	0.0376 (6)	
H2A	0.5001	0.4452	0.5982	0.045*	
H2B	0.5601	0.3596	0.5047	0.045*	
C3	0.6319 (3)	0.3779 (3)	0.6651 (4)	0.0676 (12)	
H3A	0.6208	0.4295	0.7402	0.081*	
H3B	0.7064	0.4100	0.6582	0.081*	
C4	0.6293 (3)	0.2511 (3)	0.6487 (3)	0.0465 (7)	
H4A	0.6249	0.2447	0.7186	0.056*	0.65
H4B	0.6985	0.2207	0.6256	0.056*	0.65
H4C	0.6521	0.2006	0.5793	0.056*	0.35
H4D	0.6784	0.2464	0.7143	0.056*	0.35
C5	0.5180 (4)	0.1813 (4)	0.5532 (5)	0.0455 (11)	0.65
H5A	0.5371	0.1397	0.4797	0.055*	0.65
H5B	0.4761	0.1217	0.5693	0.055*	0.65
C5B	0.5075 (7)	0.2203 (9)	0.6388 (9)	0.046 (2)	0.35
H5B1	0.4880	0.2609	0.7122	0.055*	0.35
H5B2	0.4831	0.1330	0.6084	0.055*	0.35
C6	0.2852 (4)	−0.0100 (3)	0.2908 (3)	0.0578 (9)	
H6A	0.2018	−0.0433	0.2635	0.069*	
H6B	0.3200	−0.0342	0.3493	0.069*	
C7	0.3429 (3)	−0.0522 (3)	0.1953 (3)	0.0465 (7)	
H7A	0.3031	−0.1318	0.1356	0.056*	
H7B	0.4249	−0.0558	0.2218	0.056*	
C8	0.3317 (3)	0.0430 (3)	0.1538 (3)	0.0513 (8)	
H8A	0.3889	0.0440	0.1102	0.062*	
H8B	0.2532	0.0309	0.1066	0.062*	
C9	0.3554 (3)	0.1549 (3)	0.2616 (2)	0.0432 (7)	
H9A	0.4394	0.1836	0.2935	0.052*	
H9B	0.3195	0.2185	0.2495	0.052*	
C10	−0.0179 (2)	0.0684 (2)	0.3591 (2)	0.0378 (6)	
H10A	−0.0791	0.1127	0.3866	0.045*	
H10B	0.0125	0.0328	0.4097	0.045*	
C11	−0.0631 (2)	−0.0268 (2)	0.2381 (2)	0.0406 (6)	
H11A	−0.0149	−0.0899	0.2198	0.049*	
H11B	−0.1440	−0.0637	0.2240	0.049*	
C12	−0.0541 (3)	0.0436 (3)	0.1712 (3)	0.0540 (9)	
H12A	−0.0318	−0.0047	0.1009	0.065*	
H12B	−0.1288	0.0678	0.1515	0.065*	
C13	0.0369 (3)	0.1510 (3)	0.2465 (2)	0.0454 (7)	
H13A	0.1028	0.1504	0.2122	0.054*	
H13B	0.0053	0.2249	0.2580	0.054*	
C14	0.0192 (3)	0.3867 (3)	0.5532 (3)	0.0421 (7)	
H14A	−0.0553	0.3449	0.4974	0.051*	
H14B	0.0636	0.4307	0.5232	0.051*	
C15	−0.0006 (4)	0.4690 (5)	0.6599 (3)	0.0839 (15)	
H15A	−0.0720	0.5015	0.6480	0.101*	
H15B	0.0652	0.5359	0.7031	0.101*	
C16	−0.0113 (4)	0.3935 (5)	0.7179 (4)	0.0923 (17)	
H16A	−0.0926	0.3553	0.6992	0.111*	
H16B	0.0152	0.4419	0.8005	0.111*	
C17	0.0639 (3)	0.2994 (3)	0.6771 (3)	0.0493 (8)	
H17A	0.1378	0.3196	0.7340	0.059*	
H17B	0.0240	0.2198	0.6610	0.059*	
C18	0.3477 (2)	0.7417 (2)	0.8026 (2)	0.0248 (5)	
C19	0.3067 (2)	0.7120 (2)	0.6885 (2)	0.0298 (5)	
H19A	0.2836	0.6304	0.6356	0.036*	
C20	0.2988 (2)	0.7998 (3)	0.6504 (2)	0.0370 (6)	
H20A	0.2713	0.7770	0.5722	0.044*	
C21	0.3305 (2)	0.9190 (3)	0.7249 (3)	0.0395 (6)	
H21A	0.3247	0.9783	0.6986	0.047*	
C22	0.3708 (2)	0.9512 (2)	0.8386 (3)	0.0383 (6)	
H22A	0.3921	1.0331	0.8912	0.046*	
C23	0.3800 (2)	0.8635 (2)	0.8755 (2)	0.0313 (5)	
H23A	0.4095	0.8871	0.9536	0.038*	
C24	0.2584 (2)	0.6512 (2)	0.92518 (19)	0.0254 (5)	
C25	0.1699 (2)	0.7173 (2)	0.9233 (2)	0.0337 (5)	
H25A	0.1687	0.7633	0.8823	0.040*	
C26	0.0836 (2)	0.7187 (3)	0.9790 (3)	0.0412 (6)	
H26A	0.0251	0.7650	0.9756	0.049*	
C27	0.0830 (3)	0.6528 (3)	1.0393 (3)	0.0425 (7)	
H27A	0.0247	0.6537	1.0780	0.051*	
C28	0.1687 (3)	0.5852 (3)	1.0425 (2)	0.0398 (6)	
H28A	0.1690	0.5392	1.0835	0.048*	
C29	0.2542 (2)	0.5840 (2)	0.9863 (2)	0.0318 (5)	
H29A	0.3117	0.5364	0.9891	0.038*	
C30	0.4912 (2)	0.67005 (19)	0.9294 (2)	0.0267 (5)	
C31	0.5262 (2)	0.7043 (2)	1.0449 (2)	0.0330 (6)	
H31A	0.4696	0.7137	1.0869	0.040*	
C32	0.6433 (3)	0.7251 (2)	1.1005 (2)	0.0414 (7)	
H32A	0.6646	0.7483	1.1792	0.050*	
C33	0.7263 (2)	0.7123 (2)	1.0424 (3)	0.0429 (7)	
H33A	0.8052	0.7258	1.0803	0.051*	
C34	0.6955 (2)	0.6797 (3)	0.9283 (3)	0.0424 (7)	
H34A	0.7530	0.6712	0.8874	0.051*	
C35	0.5798 (2)	0.6594 (2)	0.8735 (2)	0.0351 (6)	
H35A	0.5599	0.6373	0.7950	0.042*	

### Dichlorido(cyanotriphenylborato-κN)tetrakis(tetrahydrofuran-κO)dysprosium(III) (1-Dy) Atomic displacement parameters (Å^2^)

**Table:**

	*U*^11^	*U*^22^	*U*^33^	*U*^12^	*U*^13^	*U*^23^
Dy1	0.02183 (6)	0.01583 (6)	0.01804 (6)	0.00100 (4)	0.00370 (4)	0.00748 (4)
Cl1	0.0365 (3)	0.0313 (3)	0.0369 (3)	0.0028 (2)	0.0059 (2)	0.0243 (3)
Cl2	0.0376 (3)	0.0231 (3)	0.0322 (3)	0.0012 (2)	0.0053 (2)	0.0169 (2)
O1	0.0244 (8)	0.0269 (9)	0.0423 (10)	0.0024 (7)	0.0038 (7)	0.0090 (8)
O2	0.0381 (10)	0.0284 (9)	0.0259 (9)	0.0075 (7)	0.0121 (7)	0.0076 (7)
O3	0.0256 (8)	0.0307 (9)	0.0256 (8)	−0.0050 (7)	0.0001 (7)	0.0128 (7)
O4	0.0305 (9)	0.0291 (9)	0.0296 (9)	0.0063 (7)	0.0119 (7)	0.0136 (7)
N1	0.0272 (10)	0.0246 (10)	0.0259 (10)	0.0018 (8)	0.0032 (8)	0.0061 (8)
B1	0.0278 (12)	0.0190 (11)	0.0197 (12)	0.0026 (9)	0.0018 (10)	0.0064 (10)
C1	0.0231 (11)	0.0264 (12)	0.0236 (11)	0.0030 (9)	0.0035 (9)	0.0114 (10)
C2	0.0244 (12)	0.0460 (16)	0.0486 (16)	−0.0008 (11)	0.0057 (11)	0.0292 (14)
C3	0.0453 (19)	0.0471 (19)	0.095 (3)	−0.0098 (15)	−0.0298 (19)	0.040 (2)
C4	0.0424 (16)	0.0489 (18)	0.0525 (18)	0.0083 (13)	0.0032 (14)	0.0301 (15)
C5	0.030 (2)	0.034 (2)	0.074 (4)	0.0091 (18)	0.011 (2)	0.026 (2)
C5B	0.028 (4)	0.062 (6)	0.066 (6)	0.004 (4)	0.000 (4)	0.050 (5)
C6	0.084 (3)	0.0343 (16)	0.056 (2)	0.0188 (16)	0.0311 (19)	0.0130 (15)
C7	0.0495 (17)	0.0352 (15)	0.0406 (16)	0.0061 (13)	0.0187 (13)	0.0005 (13)
C8	0.059 (2)	0.0543 (19)	0.0323 (15)	0.0081 (16)	0.0211 (14)	0.0070 (14)
C9	0.0504 (17)	0.0402 (16)	0.0393 (15)	0.0044 (13)	0.0254 (13)	0.0123 (13)
C10	0.0328 (13)	0.0355 (14)	0.0384 (15)	−0.0075 (11)	0.0026 (11)	0.0149 (12)
C11	0.0354 (14)	0.0273 (13)	0.0456 (16)	−0.0037 (11)	−0.0010 (12)	0.0096 (12)
C12	0.061 (2)	0.0490 (18)	0.0325 (15)	−0.0120 (15)	−0.0106 (14)	0.0131 (14)
C13	0.0461 (17)	0.0491 (17)	0.0352 (15)	−0.0084 (13)	−0.0088 (12)	0.0241 (14)
C14	0.0383 (15)	0.0452 (16)	0.0558 (18)	0.0190 (13)	0.0187 (13)	0.0296 (15)
C15	0.090 (3)	0.108 (4)	0.050 (2)	0.071 (3)	0.017 (2)	0.024 (2)
C16	0.076 (3)	0.173 (5)	0.082 (3)	0.075 (3)	0.059 (3)	0.083 (4)
C17	0.0502 (18)	0.070 (2)	0.0479 (18)	0.0171 (16)	0.0252 (15)	0.0390 (17)
C18	0.0245 (11)	0.0263 (11)	0.0255 (11)	0.0049 (9)	0.0063 (9)	0.0131 (9)
C19	0.0277 (12)	0.0361 (13)	0.0260 (12)	0.0054 (10)	0.0061 (9)	0.0145 (10)
C20	0.0308 (13)	0.0594 (18)	0.0356 (14)	0.0129 (12)	0.0094 (11)	0.0335 (14)
C21	0.0312 (13)	0.0515 (17)	0.0590 (18)	0.0146 (12)	0.0155 (13)	0.0432 (16)
C22	0.0389 (14)	0.0310 (13)	0.0511 (17)	0.0080 (11)	0.0114 (13)	0.0236 (13)
C23	0.0377 (13)	0.0269 (12)	0.0300 (13)	0.0056 (10)	0.0068 (10)	0.0140 (10)
C24	0.0320 (12)	0.0189 (10)	0.0195 (10)	0.0008 (9)	0.0037 (9)	0.0047 (9)
C25	0.0355 (13)	0.0343 (13)	0.0364 (14)	0.0083 (11)	0.0094 (11)	0.0198 (12)
C26	0.0359 (14)	0.0423 (16)	0.0524 (17)	0.0136 (12)	0.0165 (13)	0.0241 (14)
C27	0.0428 (16)	0.0451 (16)	0.0454 (16)	0.0057 (13)	0.0207 (13)	0.0217 (14)
C28	0.0503 (17)	0.0371 (15)	0.0388 (15)	0.0045 (12)	0.0145 (13)	0.0224 (13)
C29	0.0403 (14)	0.0238 (12)	0.0313 (13)	0.0048 (10)	0.0096 (11)	0.0120 (10)
C30	0.0327 (12)	0.0144 (10)	0.0262 (11)	−0.0002 (9)	−0.0018 (9)	0.0071 (9)
C31	0.0423 (14)	0.0218 (11)	0.0271 (12)	0.0062 (10)	−0.0012 (10)	0.0074 (10)
C32	0.0518 (17)	0.0247 (12)	0.0311 (14)	0.0065 (12)	−0.0122 (12)	0.0065 (11)
C33	0.0317 (14)	0.0248 (13)	0.0570 (18)	0.0016 (10)	−0.0095 (13)	0.0131 (13)
C34	0.0300 (13)	0.0360 (15)	0.0563 (18)	0.0002 (11)	0.0028 (12)	0.0201 (14)
C35	0.0316 (13)	0.0351 (14)	0.0367 (14)	−0.0007 (11)	0.0016 (11)	0.0180 (12)

### Dichlorido(cyanotriphenylborato-κN)tetrakis(tetrahydrofuran-κO)dysprosium(III) (1-Dy) Geometric parameters (Å, º)

**Table:**

Dy1—O1	2.3790 (18)	C7—C8	1.519 (5)
Dy1—O2	2.3838 (17)	C8—C9	1.495 (4)
Dy1—O4	2.3932 (17)	C10—C11	1.521 (4)
Dy1—O3	2.4022 (16)	C11—C12	1.514 (4)
Dy1—N1	2.431 (2)	C12—C13	1.498 (4)
Dy1—Cl2	2.5835 (6)	C14—C15	1.456 (5)
Dy1—Cl1	2.5888 (6)	C15—C16	1.470 (7)
O1—C5	1.411 (5)	C16—C17	1.522 (5)
O1—C2	1.455 (3)	C18—C19	1.397 (3)
O1—C5B	1.597 (8)	C18—C23	1.398 (3)
O2—C9	1.454 (3)	C19—C20	1.396 (4)
O2—C6	1.476 (4)	C20—C21	1.377 (4)
O3—C13	1.439 (3)	C21—C22	1.384 (4)
O3—C10	1.455 (3)	C22—C23	1.386 (4)
O4—C14	1.445 (3)	C24—C25	1.397 (3)
O4—C17	1.465 (3)	C24—C29	1.407 (3)
N1—C1	1.141 (3)	C25—C26	1.390 (4)
B1—C1	1.621 (3)	C26—C27	1.382 (4)
B1—C18	1.625 (3)	C27—C28	1.385 (4)
B1—C24	1.632 (4)	C28—C29	1.387 (4)
B1—C30	1.641 (3)	C30—C31	1.396 (3)
C2—C3	1.505 (4)	C30—C35	1.402 (4)
C3—C4	1.507 (4)	C31—C32	1.407 (4)
C4—C5B	1.450 (9)	C32—C33	1.363 (5)
C4—C5	1.557 (6)	C33—C34	1.380 (4)
C6—C7	1.499 (4)	C34—C35	1.390 (4)
			
O1—Dy1—O2	73.01 (6)	O1—C2—C3	104.0 (2)
O1—Dy1—O4	142.46 (6)	C2—C3—C4	105.0 (3)
O2—Dy1—O4	143.59 (6)	C5B—C4—C3	98.6 (4)
O1—Dy1—O3	145.88 (6)	C3—C4—C5	103.7 (3)
O2—Dy1—O3	73.03 (6)	O1—C5—C4	105.1 (3)
O4—Dy1—O3	70.83 (6)	C4—C5B—O1	101.3 (5)
O1—Dy1—N1	73.26 (7)	O2—C6—C7	105.1 (3)
O2—Dy1—N1	144.41 (7)	C6—C7—C8	102.4 (2)
O4—Dy1—N1	71.76 (6)	C9—C8—C7	102.1 (3)
O3—Dy1—N1	140.64 (6)	O2—C9—C8	104.8 (2)
O1—Dy1—Cl2	94.03 (5)	O3—C10—C11	103.4 (2)
O2—Dy1—Cl2	85.89 (5)	C12—C11—C10	102.5 (2)
O4—Dy1—Cl2	96.37 (4)	C13—C12—C11	105.6 (2)
O3—Dy1—Cl2	86.87 (4)	O3—C13—C12	107.3 (2)
N1—Dy1—Cl2	85.47 (5)	O4—C14—C15	107.6 (3)
O1—Dy1—Cl1	87.73 (5)	C14—C15—C16	102.9 (4)
O2—Dy1—Cl1	89.32 (5)	C15—C16—C17	106.5 (3)
O4—Dy1—Cl1	85.53 (4)	O4—C17—C16	104.3 (3)
O3—Dy1—Cl1	88.55 (4)	C19—C18—C23	116.1 (2)
N1—Dy1—Cl1	100.36 (5)	C19—C18—B1	126.0 (2)
Cl2—Dy1—Cl1	174.169 (19)	C23—C18—B1	117.9 (2)
C5—O1—C2	104.8 (2)	C20—C19—C18	121.4 (2)
C2—O1—C5B	105.3 (3)	C21—C20—C19	120.8 (2)
C5—O1—Dy1	129.0 (2)	C20—C21—C22	119.2 (2)
C2—O1—Dy1	125.50 (15)	C21—C22—C23	119.7 (3)
C5B—O1—Dy1	119.5 (3)	C22—C23—C18	122.8 (2)
C9—O2—C6	108.8 (2)	C25—C24—C29	115.6 (2)
C9—O2—Dy1	127.40 (16)	C25—C24—B1	122.3 (2)
C6—O2—Dy1	123.67 (17)	C29—C24—B1	122.1 (2)
C13—O3—C10	107.00 (19)	C26—C25—C24	122.8 (2)
C13—O3—Dy1	128.45 (15)	C27—C26—C25	120.0 (3)
C10—O3—Dy1	124.48 (15)	C26—C27—C28	119.1 (3)
C14—O4—C17	107.9 (2)	C27—C28—C29	120.5 (3)
C14—O4—Dy1	120.89 (15)	C28—C29—C24	122.1 (2)
C17—O4—Dy1	126.17 (16)	C31—C30—C35	115.8 (2)
C1—N1—Dy1	163.92 (19)	C31—C30—B1	125.4 (2)
C1—B1—C18	108.07 (19)	C35—C30—B1	118.6 (2)
C1—B1—C24	105.67 (19)	C30—C31—C32	121.6 (3)
C18—B1—C24	111.50 (19)	C33—C32—C31	120.5 (3)
C1—B1—C30	106.34 (19)	C32—C33—C34	119.8 (3)
C18—B1—C30	108.93 (19)	C33—C34—C35	119.6 (3)
C24—B1—C30	115.9 (2)	C34—C35—C30	122.7 (3)
N1—C1—B1	178.7 (3)		

### Dichlorido(cyanotriphenylborato-κN)tetrakis(tetrahydrofuran-κO)ytterbium(III) (1-Y) Crystal data

**Table:**

[YCl_2_(C_19_H_15_BN)(C_4_H_8_O)_4_]	*Z* = 2
*M**_r_* = 716.35	*F*(000) = 748
Triclinic, *P*1	*D*_x_ = 1.334 Mg m^−^^3^
*a* = 12.0404 (9) Å	Mo *K*α radiation, λ = 0.71073 Å
*b* = 12.5428 (9) Å	Cell parameters from 9606 reflections
*c* = 13.4654 (10) Å	θ = 2.3–27.1°
α = 114.6967 (8)°	µ = 1.82 mm^−^^1^
β = 101.3142 (9)°	*T* = 173 K
γ = 94.3882 (9)°	Prism, colorless
*V* = 1782.7 (2) Å^3^	0.32 × 0.20 × 0.13 mm

### Dichlorido(cyanotriphenylborato-κN)tetrakis(tetrahydrofuran-κO)ytterbium(III) (1-Y) Data collection

**Table:**

Bruker SMART APEXII CCD diffractometer	7988 independent reflections
Radiation source: fine-focus sealed tube	6188 reflections with *I* > 2σ(*I*)
φ and ω scans	θ_max_ = 27.5°, θ_min_ = 1.7°
Absorption correction: multi-scan (*TWINABS*; Sheldrick, 2012)	*h* = −15→15
*T*_min_ = 0.576, *T*_max_ = 0.746	*k* = −16→14
7988 measured reflections	*l* = 0→17

### Dichlorido(cyanotriphenylborato-κN)tetrakis(tetrahydrofuran-κO)ytterbium(III) (1-Y) Refinement

**Table:**

Refinement on *F*^2^	Primary atom site location: structure-invariant direct methods
Least-squares matrix: full	Secondary atom site location: difference Fourier map
*R*[*F*^2^ > 2σ(*F*^2^)] = 0.047	Hydrogen site location: inferred from neighbouring sites
*wR*(*F*^2^) = 0.102	H-atom parameters constrained
*S* = 1.04	*w* = 1/[σ^2^(*F*_o_^2^) + (0.0438*P*)^2^ + 0.9643*P*] where *P* = (*F*_o_^2^ + 2*F*_c_^2^)/3
7988 reflections	(Δ/σ)_max_ < 0.001
425 parameters	Δρ_max_ = 0.64 e Å^−^^3^
0 restraints	Δρ_min_ = −0.80 e Å^−^^3^

### Dichlorido(cyanotriphenylborato-κN)tetrakis(tetrahydrofuran-κO)ytterbium(III) (1-Y) Special details

**Table:**

Geometry. All esds (except the esd in the dihedral angle between two l.s. planes) are estimated using the full covariance matrix. The cell esds are taken into account individually in the estimation of esds in distances, angles and torsion angles; correlations between esds in cell parameters are only used when they are defined by crystal symmetry. An approximate (isotropic) treatment of cell esds is used for estimating esds involving l.s. planes.
Refinement. A colorless crystal of approximate dimensions 0.126 x 0.198 x 0.324 mm was mounted on a glass fiber and transferred to a Bruker SMART APEX II diffractometer system. The APEX2 program package and the CELL_NOW were used to determine the unit-cell parameters. Data was collected using a 30 sec/frame scan time. The raw frame data was processed using SAINT3 and TWINABS to yield the reflection data file (HKLF5 format). Subsequent calculations were carried out using the SHELXTL program package. There were no systematic absences nor any diffraction symmetry other than the Friedel condition The centrosymmetric triclinic space group P-1 was assigned and later determined to be correct.The structure was solved by direct methods and refined on F2 by full-matrix least-squares techniques. The analytical scattering factors for neutral atoms were used throughout the analysis. Hydrogen atoms were included using a riding model. Disordered atoms were included using multiple components with partial site-occupancy-factors.Least-squares analysis yielded wR2 = 0.1023 and Goof = 1.037 for 425 variables refined against 7988 data (0.77 ), R1 = 0.0470 for those 6188 with I > 2.0sigma(I). The structure was refined as a two-component twin, BASF = 0.4868.

### Dichlorido(cyanotriphenylborato-κN)tetrakis(tetrahydrofuran-κO)ytterbium(III) (1-Y) Fractional atomic coordinates and isotropic or equivalent isotropic displacement parameters (Å^2^)

**Table:**

	*x*	*y*	*z*	*U*_iso_*/*U*_eq_	Occ. (<1)
Y1	0.24658 (3)	0.24946 (3)	0.49747 (3)	0.01941 (8)	
Cl1	0.23233 (7)	0.07981 (7)	0.55775 (7)	0.0330 (2)	
Cl2	0.25375 (7)	0.40279 (7)	0.41725 (7)	0.03054 (19)	
O1	0.44949 (18)	0.2696 (2)	0.5508 (2)	0.0338 (6)	
O2	0.30323 (19)	0.1206 (2)	0.33612 (18)	0.0304 (5)	
O3	0.07439 (18)	0.1455 (2)	0.35125 (18)	0.0291 (5)	
O4	0.08446 (18)	0.3011 (2)	0.57256 (18)	0.0291 (5)	
N1	0.3129 (2)	0.4202 (2)	0.6805 (2)	0.0273 (6)	
B1	0.3570 (3)	0.6480 (3)	0.8568 (3)	0.0242 (7)	
C1	0.3324 (3)	0.5138 (3)	0.7540 (3)	0.0249 (7)	
C2	0.5324 (3)	0.3724 (3)	0.5732 (3)	0.0368 (8)	
H2A	0.4986	0.4459	0.5980	0.044*	
H2B	0.5594	0.3611	0.5052	0.044*	
C3	0.6292 (4)	0.3788 (4)	0.6661 (4)	0.0612 (14)	
H3A	0.6173	0.4296	0.7411	0.073*	
H3B	0.7039	0.4117	0.6605	0.073*	
C4	0.6265 (3)	0.2520 (4)	0.6489 (4)	0.0481 (10)	
H4A	0.6216	0.2449	0.7185	0.058*	0.65
H4B	0.6958	0.2223	0.6263	0.058*	0.65
H4C	0.6516	0.2028	0.5806	0.058*	0.35
H4D	0.6740	0.2474	0.7154	0.058*	0.35
C5	0.5161 (5)	0.1826 (5)	0.5525 (6)	0.0425 (15)	0.65
H5A	0.5358	0.1414	0.4793	0.051*	0.65
H5B	0.4739	0.1227	0.5679	0.051*	0.65
C5B	0.5064 (10)	0.2189 (15)	0.6354 (15)	0.065 (4)	0.35
H5B1	0.4853	0.2562	0.7083	0.078*	0.35
H5B2	0.4832	0.1312	0.6023	0.078*	0.35
C6	0.2840 (4)	−0.0103 (3)	0.2910 (4)	0.0563 (12)	
H6A	0.2006	−0.0436	0.2636	0.068*	
H6B	0.3185	−0.0342	0.3500	0.068*	
C7	0.3416 (3)	−0.0537 (3)	0.1950 (3)	0.0466 (10)	
H7A	0.3008	−0.1328	0.1350	0.056*	
H7B	0.4230	−0.0582	0.2216	0.056*	
C8	0.3321 (4)	0.0423 (4)	0.1539 (3)	0.0509 (11)	
H8A	0.3900	0.0435	0.1113	0.061*	
H8B	0.2543	0.0305	0.1058	0.061*	
C9	0.3553 (3)	0.1534 (3)	0.2621 (3)	0.0435 (10)	
H9A	0.4392	0.1819	0.2949	0.052*	
H9B	0.3204	0.2171	0.2497	0.052*	
C10	−0.0181 (3)	0.0683 (3)	0.3584 (3)	0.0392 (9)	
H10A	−0.0787	0.1131	0.3859	0.047*	
H10B	0.0120	0.0329	0.4093	0.047*	
C11	−0.0638 (3)	−0.0268 (3)	0.2377 (3)	0.0417 (9)	
H11A	−0.1448	−0.0630	0.2237	0.050*	
H11B	−0.0165	−0.0904	0.2196	0.050*	
C12	−0.0539 (4)	0.0431 (4)	0.1702 (3)	0.0558 (12)	
H12A	−0.0326	−0.0059	0.0996	0.067*	
H12B	−0.1278	0.0682	0.1507	0.067*	
C13	0.0381 (3)	0.1494 (4)	0.2447 (3)	0.0469 (10)	
H13A	0.1039	0.1472	0.2102	0.056*	
H13B	0.0080	0.2236	0.2555	0.056*	
C14	0.0212 (3)	0.3876 (3)	0.5531 (3)	0.0429 (9)	
H14A	0.0648	0.4292	0.5205	0.052*	0.65
H14B	−0.0542	0.3462	0.4986	0.052*	0.65
H14C	−0.0332	0.3522	0.4774	0.052*	0.35
H14D	0.0731	0.4585	0.5633	0.052*	0.35
C15	0.0041 (7)	0.4723 (7)	0.6573 (6)	0.0430 (17)	0.65
H15A	−0.0664	0.5055	0.6446	0.052*	0.65
H15B	0.0708	0.5384	0.6986	0.052*	0.65
C16	−0.0078 (6)	0.3991 (8)	0.7203 (6)	0.0461 (17)	0.65
H16A	−0.0895	0.3649	0.7055	0.055*	0.65
H16B	0.0231	0.4485	0.8026	0.055*	0.65
C15B	−0.0431 (15)	0.4171 (17)	0.6509 (18)	0.071 (5)	0.35
H15C	−0.0002	0.4898	0.7188	0.085*	0.35
H15D	−0.1215	0.4309	0.6262	0.085*	0.35
C16B	−0.0492 (13)	0.3163 (16)	0.6758 (13)	0.061 (4)	0.35
H16C	−0.0646	0.3364	0.7505	0.073*	0.35
H16D	−0.1071	0.2475	0.6167	0.073*	0.35
C17	0.0642 (4)	0.2970 (4)	0.6745 (3)	0.0507 (11)	
H17A	0.1378	0.3126	0.7304	0.061*	0.65
H17B	0.0203	0.2185	0.6565	0.061*	0.65
H17C	0.0732	0.2184	0.6726	0.061*	0.35
H17D	0.1195	0.3598	0.7425	0.061*	0.35
C18	0.3475 (3)	0.7411 (3)	0.8012 (2)	0.0234 (6)	
C19	0.3062 (3)	0.7111 (3)	0.6868 (3)	0.0300 (7)	
H19A	0.2829	0.6295	0.6338	0.036*	
C20	0.2984 (3)	0.7984 (4)	0.6489 (3)	0.0373 (8)	
H20A	0.2706	0.7754	0.5705	0.045*	
C21	0.3304 (3)	0.9175 (4)	0.7232 (3)	0.0402 (9)	
H21A	0.3250	0.9764	0.6964	0.048*	
C22	0.3703 (3)	0.9503 (3)	0.8368 (3)	0.0377 (8)	
H22A	0.3914	1.0323	0.8893	0.045*	
C23	0.3795 (3)	0.8627 (3)	0.8743 (3)	0.0303 (7)	
H23A	0.4087	0.8865	0.9527	0.036*	
C24	0.2577 (3)	0.6505 (3)	0.9236 (3)	0.0258 (7)	
C25	0.1695 (3)	0.7157 (3)	0.9213 (3)	0.0336 (8)	
H25A	0.1685	0.7615	0.8801	0.040*	
C26	0.0831 (3)	0.7171 (4)	0.9762 (3)	0.0437 (9)	
H26A	0.0243	0.7626	0.9720	0.052*	
C27	0.0831 (3)	0.6515 (3)	1.0372 (3)	0.0435 (9)	
H27A	0.0250	0.6525	1.0761	0.052*	
C28	0.1685 (3)	0.5845 (3)	1.0411 (3)	0.0395 (9)	
H28A	0.1689	0.5390	1.0825	0.047*	
C29	0.2534 (3)	0.5837 (3)	0.9850 (3)	0.0314 (8)	
H29A	0.3108	0.5365	0.9881	0.038*	
C30	0.4906 (3)	0.6702 (3)	0.9288 (3)	0.0264 (7)	
C31	0.5250 (3)	0.7033 (3)	1.0442 (3)	0.0345 (8)	
H31A	0.4683	0.7113	1.0858	0.041*	
C32	0.6417 (3)	0.7251 (3)	1.1004 (3)	0.0446 (10)	
H32A	0.6629	0.7491	1.1795	0.053*	
C33	0.7251 (3)	0.7120 (3)	1.0424 (4)	0.0441 (10)	
H33A	0.8037	0.7245	1.0804	0.053*	
C34	0.6941 (3)	0.6806 (3)	0.9286 (4)	0.0426 (10)	
H34A	0.7511	0.6724	0.8874	0.051*	
C35	0.5787 (3)	0.6610 (3)	0.8744 (3)	0.0355 (8)	
H35A	0.5588	0.6401	0.7959	0.043*	

### Dichlorido(cyanotriphenylborato-κN)tetrakis(tetrahydrofuran-κO)ytterbium(III) (1-Y) Atomic displacement parameters (Å^2^)

**Table:**

	*U*^11^	*U*^22^	*U*^33^	*U*^12^	*U*^13^	*U*^23^
Y1	0.02100 (14)	0.01684 (13)	0.01973 (13)	0.00180 (10)	0.00476 (10)	0.00791 (10)
Cl1	0.0363 (5)	0.0321 (4)	0.0387 (5)	0.0042 (4)	0.0077 (4)	0.0244 (4)
Cl2	0.0377 (5)	0.0236 (4)	0.0332 (4)	0.0024 (3)	0.0066 (4)	0.0168 (3)
O1	0.0216 (12)	0.0270 (12)	0.0441 (14)	0.0039 (10)	0.0048 (10)	0.0092 (11)
O2	0.0347 (13)	0.0268 (13)	0.0268 (12)	0.0075 (10)	0.0124 (10)	0.0068 (10)
O3	0.0243 (12)	0.0325 (13)	0.0268 (12)	−0.0043 (10)	0.0019 (9)	0.0131 (10)
O4	0.0300 (12)	0.0300 (13)	0.0299 (12)	0.0062 (10)	0.0125 (10)	0.0133 (10)
N1	0.0262 (14)	0.0236 (14)	0.0257 (14)	0.0023 (11)	0.0047 (11)	0.0059 (12)
B1	0.0294 (19)	0.0165 (16)	0.0219 (17)	0.0023 (15)	0.0024 (15)	0.0062 (13)
C1	0.0224 (16)	0.0287 (17)	0.0249 (16)	0.0055 (13)	0.0052 (13)	0.0134 (14)
C2	0.0264 (18)	0.042 (2)	0.047 (2)	0.0009 (16)	0.0076 (16)	0.0252 (18)
C3	0.043 (2)	0.043 (3)	0.084 (3)	−0.0033 (19)	−0.019 (2)	0.032 (2)
C4	0.041 (2)	0.054 (3)	0.054 (3)	0.0083 (19)	0.0030 (19)	0.033 (2)
C5	0.026 (3)	0.036 (3)	0.065 (4)	0.009 (2)	0.009 (3)	0.024 (3)
C5B	0.023 (6)	0.099 (12)	0.108 (12)	0.001 (7)	0.002 (7)	0.086 (11)
C6	0.082 (3)	0.029 (2)	0.055 (3)	0.017 (2)	0.028 (2)	0.010 (2)
C7	0.047 (2)	0.036 (2)	0.044 (2)	0.0079 (18)	0.0209 (19)	0.0007 (18)
C8	0.058 (3)	0.053 (3)	0.032 (2)	0.008 (2)	0.0207 (19)	0.0056 (19)
C9	0.049 (2)	0.040 (2)	0.043 (2)	0.0062 (18)	0.0268 (19)	0.0123 (18)
C10	0.0304 (19)	0.039 (2)	0.043 (2)	−0.0083 (16)	0.0030 (16)	0.0193 (17)
C11	0.0314 (19)	0.0271 (18)	0.053 (2)	−0.0030 (16)	−0.0018 (17)	0.0111 (17)
C12	0.060 (3)	0.056 (3)	0.036 (2)	−0.009 (2)	−0.009 (2)	0.018 (2)
C13	0.048 (2)	0.051 (3)	0.037 (2)	−0.0078 (19)	−0.0073 (18)	0.025 (2)
C14	0.037 (2)	0.045 (2)	0.061 (3)	0.0201 (18)	0.0213 (19)	0.031 (2)
C15	0.039 (4)	0.055 (5)	0.039 (3)	0.022 (3)	0.013 (3)	0.020 (3)
C16	0.044 (4)	0.065 (5)	0.040 (4)	0.026 (4)	0.022 (3)	0.026 (4)
C15B	0.049 (10)	0.082 (14)	0.110 (15)	0.039 (9)	0.059 (11)	0.048 (13)
C16B	0.056 (9)	0.093 (12)	0.053 (9)	0.027 (9)	0.037 (7)	0.038 (9)
C17	0.051 (3)	0.076 (3)	0.049 (2)	0.020 (2)	0.029 (2)	0.041 (2)
C18	0.0194 (15)	0.0272 (16)	0.0249 (15)	0.0058 (13)	0.0066 (13)	0.0121 (13)
C19	0.0272 (18)	0.0378 (19)	0.0295 (17)	0.0084 (15)	0.0098 (14)	0.0175 (15)
C20	0.0279 (19)	0.063 (3)	0.0351 (18)	0.0153 (18)	0.0099 (16)	0.0333 (19)
C21	0.031 (2)	0.052 (2)	0.064 (3)	0.0170 (17)	0.0179 (18)	0.047 (2)
C22	0.035 (2)	0.0295 (19)	0.053 (2)	0.0081 (15)	0.0103 (17)	0.0228 (17)
C23	0.0336 (18)	0.0278 (17)	0.0301 (17)	0.0058 (15)	0.0062 (15)	0.0143 (14)
C24	0.0309 (17)	0.0204 (16)	0.0219 (16)	0.0030 (13)	0.0045 (13)	0.0065 (13)
C25	0.035 (2)	0.034 (2)	0.0369 (19)	0.0062 (16)	0.0082 (16)	0.0209 (16)
C26	0.038 (2)	0.046 (2)	0.056 (2)	0.0147 (18)	0.0178 (19)	0.0264 (19)
C27	0.043 (2)	0.048 (2)	0.046 (2)	0.0050 (19)	0.0234 (19)	0.0219 (19)
C28	0.050 (2)	0.036 (2)	0.039 (2)	0.0043 (18)	0.0143 (18)	0.0217 (17)
C29	0.038 (2)	0.0248 (18)	0.0322 (18)	0.0053 (15)	0.0121 (15)	0.0123 (15)
C30	0.0324 (18)	0.0124 (15)	0.0272 (17)	0.0005 (13)	−0.0014 (14)	0.0065 (13)
C31	0.044 (2)	0.0237 (18)	0.0276 (18)	0.0065 (15)	−0.0010 (15)	0.0077 (15)
C32	0.057 (3)	0.0235 (18)	0.0333 (19)	0.0053 (17)	−0.0145 (18)	0.0056 (15)
C33	0.033 (2)	0.0239 (19)	0.058 (3)	0.0026 (15)	−0.0088 (19)	0.0116 (18)
C34	0.033 (2)	0.033 (2)	0.058 (3)	0.0006 (16)	0.0059 (18)	0.0201 (19)
C35	0.0307 (19)	0.0341 (19)	0.040 (2)	0.0002 (15)	0.0047 (16)	0.0176 (16)

### Dichlorido(cyanotriphenylborato-κN)tetrakis(tetrahydrofuran-κO)ytterbium(III) (1-Y) Geometric parameters (Å, º)

**Table:**

Y1—O1	2.369 (2)	C10—C11	1.515 (5)
Y1—O2	2.370 (2)	C11—C12	1.518 (5)
Y1—O4	2.382 (2)	C12—C13	1.493 (5)
Y1—O3	2.390 (2)	C14—C15	1.429 (8)
Y1—N1	2.420 (3)	C14—C15B	1.583 (16)
Y1—Cl2	2.5730 (8)	C15—C16	1.503 (11)
Y1—Cl1	2.5803 (9)	C16—C17	1.578 (8)
O1—C5	1.408 (6)	C15B—C16B	1.44 (2)
O1—C2	1.452 (4)	C16B—C17	1.408 (14)
O1—C5B	1.594 (12)	C18—C19	1.396 (4)
O2—C9	1.451 (4)	C18—C23	1.396 (4)
O2—C6	1.473 (4)	C19—C20	1.390 (5)
O3—C13	1.438 (4)	C20—C21	1.376 (5)
O3—C10	1.463 (4)	C21—C22	1.377 (5)
O4—C14	1.453 (4)	C22—C23	1.391 (5)
O4—C17	1.460 (4)	C24—C25	1.392 (5)
N1—C1	1.144 (4)	C24—C29	1.407 (5)
B1—C24	1.626 (5)	C25—C26	1.388 (5)
B1—C1	1.629 (4)	C26—C27	1.384 (5)
B1—C18	1.630 (5)	C27—C28	1.383 (5)
B1—C30	1.642 (5)	C28—C29	1.384 (5)
C2—C3	1.504 (5)	C30—C35	1.388 (5)
C3—C4	1.505 (6)	C30—C31	1.393 (5)
C4—C5B	1.428 (12)	C31—C32	1.404 (5)
C4—C5	1.552 (7)	C32—C33	1.368 (6)
C6—C7	1.506 (5)	C33—C34	1.376 (6)
C7—C8	1.523 (6)	C34—C35	1.387 (5)
C8—C9	1.492 (5)		
			
O1—Y1—O2	72.94 (8)	C5B—C4—C3	99.2 (7)
O1—Y1—O4	142.45 (8)	C3—C4—C5	103.6 (4)
O2—Y1—O4	143.68 (8)	O1—C5—C4	105.3 (4)
O1—Y1—O3	145.89 (8)	C4—C5B—O1	102.3 (8)
O2—Y1—O3	73.07 (8)	O2—C6—C7	105.5 (3)
O4—Y1—O3	70.93 (8)	C6—C7—C8	102.2 (3)
O1—Y1—N1	73.27 (8)	C9—C8—C7	102.2 (3)
O2—Y1—N1	144.47 (9)	O2—C9—C8	105.4 (3)
O4—Y1—N1	71.65 (8)	O3—C10—C11	103.5 (3)
O3—Y1—N1	140.55 (9)	C10—C11—C12	102.4 (3)
O1—Y1—Cl2	93.71 (6)	C13—C12—C11	105.8 (3)
O2—Y1—Cl2	86.20 (6)	O3—C13—C12	107.4 (3)
O4—Y1—Cl2	96.39 (6)	C15—C14—O4	109.7 (4)
O3—Y1—Cl2	86.81 (6)	O4—C14—C15B	98.3 (7)
N1—Y1—Cl2	85.32 (7)	C14—C15—C16	102.8 (6)
O1—Y1—Cl1	88.01 (6)	C15—C16—C17	105.4 (5)
O2—Y1—Cl1	89.21 (6)	C16B—C15B—C14	107.1 (11)
O4—Y1—Cl1	85.42 (6)	C17—C16B—C15B	97.5 (11)
O3—Y1—Cl1	88.77 (6)	C16B—C17—O4	106.2 (7)
N1—Y1—Cl1	100.28 (7)	O4—C17—C16	103.9 (4)
Cl2—Y1—Cl1	174.39 (3)	C19—C18—C23	116.3 (3)
C5—O1—C2	104.8 (3)	C19—C18—B1	126.0 (3)
C2—O1—C5B	105.3 (5)	C23—C18—B1	117.6 (3)
C5—O1—Y1	128.8 (3)	C20—C19—C18	121.2 (3)
C2—O1—Y1	125.9 (2)	C21—C20—C19	121.0 (3)
C5B—O1—Y1	119.6 (4)	C20—C21—C22	119.3 (3)
C9—O2—C6	108.5 (3)	C21—C22—C23	119.5 (3)
C9—O2—Y1	127.3 (2)	C22—C23—C18	122.6 (3)
C6—O2—Y1	124.1 (2)	C25—C24—C29	115.5 (3)
C13—O3—C10	107.0 (2)	C25—C24—B1	122.6 (3)
C13—O3—Y1	128.5 (2)	C29—C24—B1	121.9 (3)
C10—O3—Y1	124.49 (19)	C26—C25—C24	123.2 (3)
C14—O4—C17	107.8 (3)	C27—C26—C25	119.4 (4)
C14—O4—Y1	121.12 (19)	C28—C27—C26	119.4 (3)
C17—O4—Y1	126.3 (2)	C27—C28—C29	120.2 (3)
C1—N1—Y1	164.6 (3)	C28—C29—C24	122.2 (3)
C24—B1—C1	105.7 (3)	C35—C30—C31	115.7 (3)
C24—B1—C18	111.6 (3)	C35—C30—B1	119.3 (3)
C1—B1—C18	107.8 (2)	C31—C30—B1	125.0 (3)
C24—B1—C30	116.6 (3)	C30—C31—C32	121.4 (4)
C1—B1—C30	106.0 (3)	C33—C32—C31	120.6 (3)
C18—B1—C30	108.6 (3)	C32—C33—C34	119.4 (3)
N1—C1—B1	178.4 (3)	C33—C34—C35	119.4 (4)
O1—C2—C3	103.9 (3)	C34—C35—C30	123.5 (3)
C2—C3—C4	105.0 (3)		

### Tetraethylazanium 2,2,4,6-tetraphenyl-1,3,5,2λ4,4,6-trioxatriborinan-2-ide (2) Crystal data

**Table:**

C_8_H_20_N^+^·C_24_H_20_B_3_O_3_^−^	*F*(000) = 1112
*M**_r_* = 519.08	*D*_x_ = 1.179 Mg m^−^^3^
Monoclinic, *P*2_1_/*n*	Mo *K*α radiation, λ = 0.71073 Å
*a* = 9.7245 (7) Å	Cell parameters from 9884 reflections
*b* = 18.3021 (13) Å	θ = 2.4–28.3°
*c* = 16.7716 (12) Å	µ = 0.07 mm^−^^1^
β = 101.5456 (9)°	*T* = 133 K
*V* = 2924.6 (4) Å^3^	Prism, colorless
*Z* = 4	0.37 × 0.33 × 0.23 mm

### Tetraethylazanium 2,2,4,6-tetraphenyl-1,3,5,2λ4,4,6-trioxatriborinan-2-ide (2) Data collection

**Table:**

Bruker SMART APEXII CCD diffractometer	5030 reflections with *I* > 2σ(*I*)
Radiation source: fine-focus sealed tube	*R*_int_ = 0.029
φ and ω scans	θ_max_ = 26.4°, θ_min_ = 1.7°
Absorption correction: multi-scan (SADABS; Krause et al., 2015)	*h* = −12→12
*T*_min_ = 0.834, *T*_max_ = 0.862	*k* = −22→22
31741 measured reflections	*l* = −20→20
5959 independent reflections	

### Tetraethylazanium 2,2,4,6-tetraphenyl-1,3,5,2λ4,4,6-trioxatriborinan-2-ide (2) Refinement

**Table:**

Refinement on *F*^2^	Primary atom site location: dual space
Least-squares matrix: full	Secondary atom site location: difference Fourier map
*R*[*F*^2^ > 2σ(*F*^2^)] = 0.064	Hydrogen site location: inferred from neighbouring sites
*wR*(*F*^2^) = 0.179	H-atom parameters constrained
*S* = 1.02	*w* = 1/[σ^2^(*F*_o_^2^) + (0.0909*P*)^2^ + 2.9541*P*] where *P* = (*F*_o_^2^ + 2*F*_c_^2^)/3
5959 reflections	(Δ/σ)_max_ = 0.001
442 parameters	Δρ_max_ = 1.06 e Å^−^^3^
0 restraints	Δρ_min_ = −0.47 e Å^−^^3^

### Tetraethylazanium 2,2,4,6-tetraphenyl-1,3,5,2λ4,4,6-trioxatriborinan-2-ide (2) Special details

**Table:**

Geometry. All esds (except the esd in the dihedral angle between two l.s. planes) are estimated using the full covariance matrix. The cell esds are taken into account individually in the estimation of esds in distances, angles and torsion angles; correlations between esds in cell parameters are only used when they are defined by crystal symmetry. An approximate (isotropic) treatment of cell esds is used for estimating esds involving l.s. planes.
Refinement. A colorless crystal of approximate dimensions 0.228 x 0.331 x 0.367 mm was mounted on a glass fiber and transferred to a Bruker SMART APEX II diffractometer. The APEX2 program package was used to determine the unit-cell parameters and for data collection (20 sec/frame scan time for a sphere of diffraction data). The raw frame data was processed using SAINT and SADABS to yield the reflection data file. Subsequent calculations were carried out using the SHELXTL program. The diffraction symmetry was 2/m and the systematic absences were consistent with the monoclinic space group P21/n that was later determined to be correct.The structure was solved by dual space methods and refined on F2 by full-matrix least-squares techniques. The analytical scattering factors for neutral atoms were used throughout the analysis. Hydrogen atoms were included using a riding model. The tetraethylammonium ion was fully disordered. The disordered atoms were included using multiple components with partial site-occupancy-factors.Least-squares analysis yielded wR2 = 0.1785 and Goof = 1.017 for 442 variables refined against 5959 data (0.80 Å), R1 = 0.0639 for those 5030 data with I > 2.0sigma(I).

### Tetraethylazanium 2,2,4,6-tetraphenyl-1,3,5,2λ4,4,6-trioxatriborinan-2-ide (2) Fractional atomic coordinates and isotropic or equivalent isotropic displacement parameters (Å^2^)

**Table:**

	*x*	*y*	*z*	*U*_iso_*/*U*_eq_	Occ. (<1)
B1	0.4762 (2)	0.56789 (11)	0.77924 (12)	0.0193 (4)	
B2	0.6190 (2)	0.62127 (11)	0.68613 (12)	0.0177 (4)	
B3	0.6440 (2)	0.66930 (11)	0.82091 (13)	0.0190 (4)	
O1	0.52579 (14)	0.57190 (7)	0.69997 (7)	0.0215 (3)	
O2	0.67303 (14)	0.67482 (7)	0.74274 (8)	0.0226 (3)	
O3	0.56271 (14)	0.61696 (7)	0.84114 (7)	0.0212 (3)	
C1	0.3139 (2)	0.59569 (10)	0.76601 (11)	0.0208 (4)	
C2	0.2638 (2)	0.63277 (11)	0.82703 (12)	0.0257 (4)	
H2A	0.3257	0.6410	0.8777	0.031*	
C3	0.1269 (2)	0.65811 (11)	0.81642 (13)	0.0295 (5)	
H3A	0.0968	0.6832	0.8595	0.035*	
C4	0.0340 (2)	0.64703 (12)	0.74362 (14)	0.0310 (5)	
H4A	−0.0598	0.6644	0.7361	0.037*	
C5	0.0796 (2)	0.61020 (14)	0.68181 (14)	0.0384 (5)	
H5A	0.0170	0.6021	0.6314	0.046*	
C6	0.2174 (2)	0.58499 (13)	0.69332 (13)	0.0318 (5)	
H6A	0.2467	0.5596	0.6502	0.038*	
C7	0.49230 (18)	0.48395 (10)	0.81153 (11)	0.0191 (4)	
C8	0.5228 (2)	0.46709 (11)	0.89406 (12)	0.0291 (5)	
H8A	0.5386	0.5058	0.9326	0.035*	
C9	0.5309 (3)	0.39533 (13)	0.92183 (14)	0.0368 (5)	
H9A	0.5528	0.3856	0.9786	0.044*	
C10	0.5071 (2)	0.33809 (11)	0.86710 (14)	0.0325 (5)	
H10A	0.5101	0.2891	0.8859	0.039*	
C11	0.4790 (2)	0.35292 (11)	0.78472 (14)	0.0295 (5)	
H11A	0.4645	0.3139	0.7466	0.035*	
C12	0.4719 (2)	0.42482 (11)	0.75758 (12)	0.0245 (4)	
H12A	0.4525	0.4341	0.7007	0.029*	
C13	0.67346 (18)	0.62034 (10)	0.60354 (11)	0.0189 (4)	
C14	0.6536 (2)	0.55854 (11)	0.55359 (12)	0.0253 (4)	
H14A	0.6069	0.5172	0.5698	0.030*	
C15	0.7006 (3)	0.55642 (13)	0.48118 (13)	0.0368 (5)	
H15A	0.6873	0.5136	0.4485	0.044*	
C16	0.7671 (3)	0.61664 (15)	0.45617 (14)	0.0402 (6)	
H16A	0.7992	0.6152	0.4063	0.048*	
C17	0.7867 (2)	0.67887 (14)	0.50387 (13)	0.0368 (5)	
H17A	0.8312	0.7205	0.4865	0.044*	
C18	0.7410 (2)	0.68035 (12)	0.57715 (12)	0.0265 (4)	
H18A	0.7560	0.7230	0.6100	0.032*	
C19	0.71090 (19)	0.72806 (10)	0.88572 (11)	0.0215 (4)	
C20	0.8255 (2)	0.77103 (11)	0.87687 (13)	0.0275 (4)	
H20A	0.8646	0.7654	0.8297	0.033*	
C21	0.8835 (2)	0.82185 (12)	0.93569 (15)	0.0358 (5)	
H21A	0.9633	0.8495	0.9293	0.043*	
C22	0.8252 (2)	0.83221 (12)	1.00344 (14)	0.0336 (5)	
H22A	0.8639	0.8674	1.0433	0.040*	
C23	0.7105 (2)	0.79122 (12)	1.01311 (13)	0.0335 (5)	
H23A	0.6695	0.7985	1.0594	0.040*	
C24	0.6552 (2)	0.73938 (12)	0.95518 (12)	0.0289 (4)	
H24A	0.5773	0.7109	0.9630	0.035*	
N4	0.4859 (11)	0.8966 (7)	0.6646 (6)	0.0247 (17)	0.567 (3)
C25	0.4659 (5)	0.9730 (2)	0.6325 (4)	0.0550 (15)	0.567 (3)
H25A	0.5493	0.9879	0.6111	0.066*	0.567 (3)
H25B	0.4569	1.0065	0.6775	0.066*	0.567 (3)
C26	0.3277 (5)	0.9795 (3)	0.5612 (3)	0.0479 (12)	0.567 (3)
H26A	0.3213	1.0291	0.5387	0.072*	0.567 (3)
H26B	0.2441	0.9691	0.5835	0.072*	0.567 (3)
H26C	0.3339	0.9443	0.5180	0.072*	0.567 (3)
C27	0.4859 (6)	0.8388 (3)	0.5960 (3)	0.0553 (13)	0.567 (3)
H27A	0.5212	0.7923	0.6225	0.066*	0.567 (3)
H27D	0.3871	0.8306	0.5686	0.066*	0.567 (3)
C28	0.5623 (8)	0.8535 (3)	0.5362 (3)	0.0687 (18)	0.567 (3)
H28A	0.5610	0.8106	0.5011	0.103*	0.567 (3)
H28B	0.6595	0.8651	0.5618	0.103*	0.567 (3)
H28C	0.5204	0.8952	0.5034	0.103*	0.567 (3)
C29	0.6298 (5)	0.8933 (3)	0.7230 (5)	0.082 (3)	0.567 (3)
H29A	0.6458	0.8425	0.7431	0.098*	0.567 (3)
H29D	0.7033	0.9047	0.6917	0.098*	0.567 (3)
C30	0.6475 (10)	0.9406 (5)	0.7909 (5)	0.114 (4)	0.567 (3)
H30A	0.7443	0.9374	0.8214	0.170*	0.567 (3)
H30B	0.5829	0.9263	0.8262	0.170*	0.567 (3)
H30C	0.6273	0.9909	0.7722	0.170*	0.567 (3)
C31	0.3724 (4)	0.8759 (3)	0.7034 (3)	0.0452 (12)	0.567 (3)
H31A	0.2815	0.8906	0.6692	0.054*	0.567 (3)
H31B	0.3825	0.9007	0.7568	0.054*	0.567 (3)
C32	0.3769 (6)	0.7839 (3)	0.7165 (4)	0.0343 (11)	0.567 (3)
H32A	0.2809	0.7649	0.7076	0.051*	0.567 (3)
H32B	0.4257	0.7723	0.7720	0.051*	0.567 (3)
H32C	0.4268	0.7615	0.6774	0.051*	0.567 (3)
N4B	0.4989 (18)	0.9025 (10)	0.6867 (8)	0.030 (3)	0.433 (3)
C25B	0.6237 (6)	0.9310 (3)	0.6647 (4)	0.0357 (12)	0.433 (3)
H25C	0.6315	0.9100	0.6114	0.043*	0.433 (3)
H25D	0.7057	0.9136	0.7052	0.043*	0.433 (3)
C26B	0.6326 (4)	1.0148 (2)	0.6590 (3)	0.0206 (9)	0.433 (3)
H26D	0.7147	1.0282	0.6365	0.031*	0.433 (3)
H26E	0.6415	1.0361	0.7134	0.031*	0.433 (3)
H26F	0.5474	1.0334	0.6235	0.031*	0.433 (3)
C27B	0.3630 (5)	0.9241 (3)	0.6138 (3)	0.0358 (12)	0.433 (3)
H27B	0.2776	0.9014	0.6265	0.043*	0.433 (3)
H27C	0.3499	0.9778	0.6139	0.043*	0.433 (3)
C28B	0.3771 (8)	0.9007 (4)	0.5299 (4)	0.0544 (17)	0.433 (3)
H28D	0.2900	0.9117	0.4911	0.082*	0.433 (3)
H28E	0.3954	0.8481	0.5297	0.082*	0.433 (3)
H28F	0.4552	0.9271	0.5141	0.082*	0.433 (3)
C29B	0.4530 (6)	0.9384 (3)	0.7578 (3)	0.0360 (12)	0.433 (3)
H29B	0.3618	0.9178	0.7644	0.043*	0.433 (3)
H29C	0.4403	0.9915	0.7474	0.043*	0.433 (3)
C30B	0.5623 (6)	0.9263 (3)	0.8356 (3)	0.0295 (11)	0.433 (3)
H30D	0.5333	0.9518	0.8809	0.044*	0.433 (3)
H30E	0.6532	0.9453	0.8284	0.044*	0.433 (3)
H30F	0.5707	0.8738	0.8476	0.044*	0.433 (3)
C31B	0.4989 (5)	0.8204 (3)	0.6900 (3)	0.0312 (11)	0.433 (3)
H31C	0.5934	0.8007	0.7127	0.037*	0.433 (3)
H31D	0.4633	0.7986	0.6358	0.037*	0.433 (3)
C32B	0.3869 (8)	0.8069 (5)	0.7537 (5)	0.050 (2)	0.433 (3)
H32D	0.3422	0.7590	0.7430	0.075*	0.433 (3)
H32E	0.3148	0.8451	0.7448	0.075*	0.433 (3)
H32F	0.4387	0.8089	0.8101	0.075*	0.433 (3)

### Tetraethylazanium 2,2,4,6-tetraphenyl-1,3,5,2λ4,4,6-trioxatriborinan-2-ide (2) Atomic displacement parameters (Å^2^)

**Table:**

	*U*^11^	*U*^22^	*U*^33^	*U*^12^	*U*^13^	*U*^23^
B1	0.0231 (10)	0.0195 (10)	0.0164 (9)	−0.0042 (8)	0.0062 (8)	−0.0017 (7)
B2	0.0160 (9)	0.0192 (10)	0.0177 (9)	0.0028 (7)	0.0030 (7)	0.0022 (7)
B3	0.0163 (9)	0.0193 (10)	0.0209 (10)	0.0016 (7)	0.0023 (8)	−0.0002 (8)
O1	0.0255 (7)	0.0231 (7)	0.0169 (6)	−0.0053 (5)	0.0065 (5)	−0.0020 (5)
O2	0.0242 (7)	0.0237 (7)	0.0217 (7)	−0.0065 (5)	0.0089 (5)	−0.0046 (5)
O3	0.0249 (7)	0.0210 (7)	0.0177 (6)	−0.0045 (5)	0.0047 (5)	−0.0023 (5)
C1	0.0258 (9)	0.0151 (8)	0.0224 (9)	−0.0025 (7)	0.0069 (7)	0.0022 (7)
C2	0.0303 (10)	0.0269 (10)	0.0198 (9)	0.0009 (8)	0.0049 (8)	0.0004 (7)
C3	0.0332 (11)	0.0280 (10)	0.0301 (11)	0.0064 (8)	0.0132 (9)	0.0000 (8)
C4	0.0254 (10)	0.0293 (11)	0.0389 (12)	0.0042 (8)	0.0080 (9)	0.0007 (9)
C5	0.0301 (11)	0.0470 (14)	0.0342 (12)	0.0054 (10)	−0.0028 (9)	−0.0108 (10)
C6	0.0288 (11)	0.0397 (12)	0.0263 (10)	0.0044 (9)	0.0037 (8)	−0.0099 (9)
C7	0.0152 (8)	0.0220 (9)	0.0205 (9)	−0.0023 (7)	0.0047 (7)	−0.0009 (7)
C8	0.0378 (11)	0.0272 (10)	0.0217 (10)	−0.0039 (9)	0.0041 (8)	0.0009 (8)
C9	0.0447 (13)	0.0349 (12)	0.0286 (11)	−0.0009 (10)	0.0022 (10)	0.0118 (9)
C10	0.0288 (11)	0.0208 (10)	0.0489 (13)	0.0039 (8)	0.0101 (10)	0.0108 (9)
C11	0.0286 (10)	0.0204 (10)	0.0429 (12)	−0.0007 (8)	0.0151 (9)	−0.0064 (8)
C12	0.0276 (10)	0.0233 (9)	0.0238 (9)	−0.0027 (8)	0.0080 (8)	−0.0019 (7)
C13	0.0143 (8)	0.0241 (9)	0.0179 (8)	0.0021 (7)	0.0022 (7)	0.0015 (7)
C14	0.0288 (10)	0.0248 (10)	0.0229 (9)	0.0035 (8)	0.0062 (8)	0.0000 (7)
C15	0.0431 (13)	0.0420 (13)	0.0273 (11)	0.0055 (10)	0.0118 (10)	−0.0083 (9)
C16	0.0364 (12)	0.0659 (17)	0.0220 (10)	−0.0008 (11)	0.0149 (9)	−0.0032 (10)
C17	0.0315 (11)	0.0531 (14)	0.0275 (11)	−0.0125 (10)	0.0097 (9)	0.0066 (10)
C18	0.0252 (10)	0.0309 (10)	0.0233 (10)	−0.0055 (8)	0.0046 (8)	0.0008 (8)
C19	0.0201 (9)	0.0212 (9)	0.0224 (9)	0.0005 (7)	0.0021 (7)	−0.0009 (7)
C20	0.0226 (10)	0.0277 (10)	0.0332 (11)	−0.0030 (8)	0.0082 (8)	−0.0070 (8)
C21	0.0248 (10)	0.0324 (12)	0.0499 (14)	−0.0088 (9)	0.0071 (10)	−0.0133 (10)
C22	0.0323 (11)	0.0293 (11)	0.0355 (12)	−0.0028 (9)	−0.0020 (9)	−0.0144 (9)
C23	0.0409 (12)	0.0339 (12)	0.0257 (10)	−0.0037 (9)	0.0071 (9)	−0.0090 (9)
C24	0.0317 (11)	0.0297 (11)	0.0259 (10)	−0.0084 (8)	0.0071 (8)	−0.0052 (8)
N4	0.016 (2)	0.026 (3)	0.030 (5)	−0.0004 (19)	−0.001 (3)	0.001 (3)
C25	0.044 (3)	0.020 (2)	0.115 (4)	0.0086 (17)	0.048 (3)	0.020 (2)
C26	0.048 (3)	0.048 (3)	0.049 (3)	0.011 (2)	0.013 (2)	0.007 (2)
C27	0.066 (3)	0.042 (3)	0.057 (3)	0.011 (2)	0.009 (3)	−0.010 (2)
C28	0.115 (5)	0.052 (3)	0.038 (3)	0.022 (3)	0.014 (3)	0.004 (2)
C29	0.031 (3)	0.030 (3)	0.162 (7)	0.010 (2)	−0.032 (3)	−0.018 (3)
C30	0.108 (7)	0.106 (6)	0.093 (6)	0.054 (5)	−0.060 (5)	−0.043 (5)
C31	0.0221 (19)	0.057 (3)	0.058 (3)	0.0066 (18)	0.0125 (18)	0.037 (2)
C32	0.031 (2)	0.026 (2)	0.048 (3)	−0.0033 (18)	0.013 (2)	0.006 (2)
N4B	0.044 (5)	0.018 (3)	0.027 (6)	−0.007 (3)	0.005 (4)	−0.002 (4)
C25B	0.028 (3)	0.026 (3)	0.054 (3)	0.004 (2)	0.011 (2)	0.007 (2)
C26B	0.0155 (19)	0.015 (2)	0.033 (2)	−0.0087 (15)	0.0080 (17)	−0.0005 (16)
C27B	0.025 (2)	0.036 (3)	0.042 (3)	0.001 (2)	−0.001 (2)	0.011 (2)
C28B	0.061 (4)	0.057 (4)	0.039 (3)	−0.017 (3)	−0.004 (3)	0.002 (3)
C29B	0.036 (3)	0.027 (3)	0.045 (3)	−0.001 (2)	0.010 (2)	−0.006 (2)
C30B	0.036 (3)	0.029 (2)	0.022 (2)	−0.004 (2)	0.002 (2)	0.0007 (19)
C31B	0.027 (2)	0.021 (2)	0.045 (3)	0.0010 (18)	0.005 (2)	−0.001 (2)
C32B	0.043 (4)	0.048 (5)	0.066 (5)	0.021 (4)	0.028 (4)	0.036 (4)

### Tetraethylazanium 2,2,4,6-tetraphenyl-1,3,5,2λ4,4,6-trioxatriborinan-2-ide (2) Geometric parameters (Å, º)

**Table:**

B1—O3	1.497 (2)	C15—C16	1.385 (4)
B1—O1	1.504 (2)	C16—C17	1.383 (4)
B1—C7	1.626 (3)	C17—C18	1.388 (3)
B1—C1	1.631 (3)	C19—C24	1.395 (3)
B2—O1	1.333 (2)	C19—C20	1.396 (3)
B2—O2	1.392 (2)	C20—C21	1.391 (3)
B2—C13	1.579 (3)	C21—C22	1.380 (3)
B3—O3	1.329 (2)	C22—C23	1.380 (3)
B3—O2	1.399 (2)	C23—C24	1.387 (3)
B3—C19	1.574 (3)	N4—C31	1.441 (13)
C1—C2	1.394 (3)	N4—C25	1.497 (14)
C1—C6	1.395 (3)	N4—C29	1.541 (11)
C2—C3	1.388 (3)	N4—C27	1.561 (11)
C3—C4	1.381 (3)	C25—C26	1.615 (8)
C4—C5	1.382 (3)	C27—C28	1.390 (8)
C5—C6	1.394 (3)	C29—C30	1.414 (9)
C7—C8	1.391 (3)	C31—C32	1.697 (7)
C7—C12	1.399 (3)	N4B—C25B	1.435 (19)
C8—C9	1.391 (3)	N4B—C31B	1.504 (18)
C9—C10	1.381 (3)	N4B—C29B	1.506 (15)
C10—C11	1.381 (3)	N4B—C27B	1.659 (16)
C11—C12	1.390 (3)	C25B—C26B	1.541 (6)
C13—C18	1.396 (3)	C27B—C28B	1.504 (9)
C13—C14	1.398 (3)	C29B—C30B	1.525 (8)
C14—C15	1.381 (3)	C31B—C32B	1.688 (9)
			
O3—B1—O1	110.41 (14)	C15—C14—C13	121.3 (2)
O3—B1—C7	109.43 (15)	C14—C15—C16	120.0 (2)
O1—B1—C7	108.31 (15)	C17—C16—C15	119.9 (2)
O3—B1—C1	107.53 (15)	C16—C17—C18	119.8 (2)
O1—B1—C1	109.70 (15)	C17—C18—C13	121.3 (2)
C7—B1—C1	111.47 (15)	C24—C19—C20	117.19 (18)
O1—B2—O2	122.37 (16)	C24—C19—B3	120.14 (17)
O1—B2—C13	120.31 (17)	C20—C19—B3	122.67 (17)
O2—B2—C13	117.32 (16)	C21—C20—C19	121.36 (19)
O3—B3—O2	122.23 (17)	C22—C21—C20	120.1 (2)
O3—B3—C19	120.06 (17)	C23—C22—C21	119.76 (19)
O2—B3—C19	117.70 (16)	C22—C23—C24	119.9 (2)
B2—O1—B1	122.45 (15)	C23—C24—C19	121.68 (19)
B2—O2—B3	117.97 (15)	C31—N4—C25	110.6 (8)
B3—O3—B1	122.39 (15)	C31—N4—C29	112.5 (8)
C2—C1—C6	115.91 (18)	C25—N4—C29	107.5 (8)
C2—C1—B1	121.51 (17)	C31—N4—C27	105.6 (8)
C6—C1—B1	122.57 (17)	C25—N4—C27	112.8 (7)
C3—C2—C1	122.39 (19)	C29—N4—C27	107.9 (8)
C4—C3—C2	120.37 (19)	N4—C25—C26	111.4 (5)
C3—C4—C5	118.9 (2)	C28—C27—N4	118.7 (7)
C4—C5—C6	120.1 (2)	C30—C29—N4	115.9 (6)
C5—C6—C1	122.3 (2)	N4—C31—C32	108.4 (6)
C8—C7—C12	116.51 (18)	C25B—N4B—C31B	112.3 (12)
C8—C7—B1	121.88 (17)	C25B—N4B—C29B	116.4 (10)
C12—C7—B1	121.58 (16)	C31B—N4B—C29B	113.9 (11)
C9—C8—C7	122.0 (2)	C25B—N4B—C27B	108.2 (10)
C10—C9—C8	120.2 (2)	C31B—N4B—C27B	105.1 (9)
C11—C10—C9	119.29 (19)	C29B—N4B—C27B	99.2 (11)
C10—C11—C12	120.06 (19)	N4B—C25B—C26B	116.0 (8)
C11—C12—C7	121.96 (19)	C28B—C27B—N4B	114.4 (7)
C18—C13—C14	117.63 (17)	N4B—C29B—C30B	110.3 (7)
C18—C13—B2	122.09 (17)	N4B—C31B—C32B	100.0 (8)
C14—C13—B2	120.28 (17)		

### Tetraethylazanium cyanodiphenyl-λ4-boranyl diphenylborinate (3) Crystal data

**Table:**

C_8_H_20_N^+^·C_25_H_20_B_2_NO^−^	*F*(000) = 1080
*M**_r_* = 502.29	*D*_x_ = 1.172 Mg m^−^^3^
Monoclinic, *P*2_1_/*n*	Mo *K*α radiation, λ = 0.71073 Å
*a* = 11.0269 (8) Å	Cell parameters from 9716 reflections
*b* = 13.9387 (11) Å	θ = 2.2–29.1°
*c* = 18.8488 (14) Å	µ = 0.07 mm^−^^1^
β = 100.6357 (10)°	*T* = 88 K
*V* = 2847.3 (4) Å^3^	Irregular, colorless
*Z* = 4	0.28 × 0.27 × 0.22 mm

### Tetraethylazanium cyanodiphenyl-λ4-boranyl diphenylborinate (3) Data collection

**Table:**

Bruker SMART APEXII CCD diffractometer	5500 reflections with *I* > 2σ(*I*)
Radiation source: fine-focus sealed tube	*R*_int_ = 0.042
φ and ω scans	θ_max_ = 29.1°, θ_min_ = 1.8°
Absorption correction: multi-scan (SADABS; Krause et al., 2015)	*h* = −14→14
*T*_min_ = 0.715, *T*_max_ = 0.746	*k* = −18→18
34768 measured reflections	*l* = −25→25
7242 independent reflections	

### Tetraethylazanium cyanodiphenyl-λ4-boranyl diphenylborinate (3) Refinement

**Table:**

Refinement on *F*^2^	Primary atom site location: dual space
Least-squares matrix: full	Secondary atom site location: difference Fourier map
*R*[*F*^2^ > 2σ(*F*^2^)] = 0.048	Hydrogen site location: inferred from neighbouring sites
*wR*(*F*^2^) = 0.119	H-atom parameters constrained
*S* = 1.04	*w* = 1/[σ^2^(*F*_o_^2^) + (0.0477*P*)^2^ + 1.1994*P*] where *P* = (*F*_o_^2^ + 2*F*_c_^2^)/3
7242 reflections	(Δ/σ)_max_ < 0.001
347 parameters	Δρ_max_ = 0.41 e Å^−^^3^
0 restraints	Δρ_min_ = −0.24 e Å^−^^3^

### Tetraethylazanium cyanodiphenyl-λ4-boranyl diphenylborinate (3) Special details

**Table:**

Geometry. All esds (except the esd in the dihedral angle between two l.s. planes) are estimated using the full covariance matrix. The cell esds are taken into account individually in the estimation of esds in distances, angles and torsion angles; correlations between esds in cell parameters are only used when they are defined by crystal symmetry. An approximate (isotropic) treatment of cell esds is used for estimating esds involving l.s. planes.
Refinement. A colorless crystal of approximate dimensions 0.216 x 0.265 x 0.280 mm was mounted in a cryoloop and transferred to a Bruker SMART APEX II diffractometer. The APEX2 program package was used to determine the unit-cell parameters and for data collection (30 sec/frame scan time for a sphere of diffraction data). The raw frame data was processed using SAINT and SADABS to yield the reflection data file. Subsequent calculations were carried out using the SHELXTL program. The diffraction symmetry was 2/m and the systematic absences were consistent with the monoclinic space group P21/n that was later determined to be correct.The structure was solved by dual space methods and refined on F2 by full-matrix least-squares techniques. The analytical scattering factors for neutral atoms were used throughout the analysis. Hydrogen atoms were included using a riding model.Least-squares analysis yielded wR2 = 0.1188 and Goof = 1.038 for 347 variables refined against 7242 data (0.73 Å), R1 = 0.0476 for those 5500 data with I > 2.0sigma(I).

### Tetraethylazanium cyanodiphenyl-λ4-boranyl diphenylborinate (3) Fractional atomic coordinates and isotropic or equivalent isotropic displacement parameters (Å^2^)

**Table:**

	*x*	*y*	*z*	*U*_iso_*/*U*_eq_	
O1	0.54584 (8)	0.23892 (7)	0.71613 (5)	0.0169 (2)	
N1	0.65451 (11)	0.48002 (9)	0.69950 (7)	0.0223 (3)	
B1	0.57013 (13)	0.29746 (11)	0.65455 (8)	0.0150 (3)	
B2	0.48921 (13)	0.24852 (11)	0.77251 (8)	0.0164 (3)	
C1	0.61696 (12)	0.40504 (10)	0.68153 (7)	0.0164 (3)	
C2	0.68248 (12)	0.24787 (9)	0.62196 (7)	0.0155 (3)	
C3	0.74007 (12)	0.29439 (10)	0.57114 (7)	0.0167 (3)	
H3	0.7132	0.3569	0.5555	0.020*	
C4	0.83524 (13)	0.25223 (10)	0.54282 (7)	0.0193 (3)	
H4	0.8716	0.2856	0.5081	0.023*	
C5	0.87694 (13)	0.16128 (10)	0.56543 (8)	0.0229 (3)	
H5	0.9419	0.1321	0.5463	0.028*	
C6	0.82301 (14)	0.11343 (11)	0.61610 (9)	0.0271 (3)	
H6	0.8516	0.0515	0.6322	0.033*	
C7	0.72688 (13)	0.15612 (10)	0.64344 (8)	0.0229 (3)	
H7	0.6903	0.1219	0.6777	0.028*	
C8	0.44546 (12)	0.30775 (9)	0.59338 (7)	0.0155 (3)	
C9	0.42486 (12)	0.38370 (10)	0.54401 (7)	0.0178 (3)	
H9	0.4882	0.4299	0.5440	0.021*	
C10	0.31446 (13)	0.39366 (10)	0.49485 (7)	0.0210 (3)	
H10	0.3036	0.4461	0.4621	0.025*	
C11	0.22064 (13)	0.32728 (11)	0.49356 (7)	0.0243 (3)	
H11	0.1445	0.3347	0.4610	0.029*	
C12	0.23942 (14)	0.24983 (12)	0.54039 (8)	0.0279 (3)	
H12	0.1765	0.2030	0.5392	0.033*	
C13	0.35024 (13)	0.24034 (11)	0.58922 (8)	0.0234 (3)	
H13	0.3615	0.1865	0.6206	0.028*	
C14	0.42967 (12)	0.34557 (10)	0.79522 (7)	0.0179 (3)	
C15	0.35810 (12)	0.40696 (10)	0.74516 (7)	0.0205 (3)	
H15	0.3443	0.3909	0.6953	0.025*	
C16	0.30703 (13)	0.49057 (10)	0.76672 (8)	0.0234 (3)	
H16	0.2571	0.5299	0.7318	0.028*	
C17	0.32839 (13)	0.51700 (10)	0.83869 (8)	0.0240 (3)	
H17	0.2951	0.5752	0.8531	0.029*	
C18	0.39858 (13)	0.45811 (10)	0.88957 (8)	0.0228 (3)	
H18	0.4135	0.4757	0.9391	0.027*	
C19	0.44713 (13)	0.37332 (10)	0.86811 (7)	0.0195 (3)	
H19	0.4935	0.3329	0.9037	0.023*	
C20	0.48423 (12)	0.15502 (9)	0.82032 (7)	0.0160 (3)	
C21	0.39985 (12)	0.14284 (10)	0.86704 (7)	0.0171 (3)	
H21	0.3452	0.1939	0.8725	0.021*	
C22	0.39383 (13)	0.05835 (10)	0.90564 (7)	0.0199 (3)	
H22	0.3345	0.0515	0.9360	0.024*	
C23	0.47508 (13)	−0.01578 (10)	0.89946 (7)	0.0204 (3)	
H23	0.4719	−0.0735	0.9259	0.024*	
C24	0.56085 (13)	−0.00558 (10)	0.85476 (7)	0.0210 (3)	
H24	0.6171	−0.0561	0.8510	0.025*	
C25	0.56500 (13)	0.07811 (10)	0.81549 (7)	0.0187 (3)	
H25	0.6237	0.0837	0.7846	0.022*	
N2	0.51337 (10)	0.80126 (8)	0.65697 (6)	0.0166 (2)	
C26	0.53989 (13)	0.69500 (10)	0.65074 (8)	0.0228 (3)	
H26A	0.5995	0.6869	0.6178	0.027*	
H26B	0.5797	0.6709	0.6988	0.027*	
C27	0.42736 (13)	0.63381 (11)	0.62331 (8)	0.0253 (3)	
H27A	0.3910	0.6533	0.5740	0.038*	
H27B	0.3666	0.6424	0.6547	0.038*	
H27C	0.4517	0.5662	0.6235	0.038*	
C28	0.42039 (13)	0.81887 (10)	0.70616 (7)	0.0206 (3)	
H28A	0.4178	0.8886	0.7159	0.025*	
H28B	0.3376	0.7998	0.6801	0.025*	
C29	0.44632 (14)	0.76619 (11)	0.77769 (8)	0.0252 (3)	
H29A	0.4397	0.6969	0.7690	0.038*	
H29B	0.3862	0.7859	0.8072	0.038*	
H29C	0.5298	0.7817	0.8031	0.038*	
C30	0.63326 (12)	0.85169 (10)	0.68896 (7)	0.0200 (3)	
H30A	0.6145	0.9194	0.6986	0.024*	
H30B	0.6676	0.8214	0.7359	0.024*	
C31	0.73123 (13)	0.84982 (12)	0.64196 (8)	0.0256 (3)	
H31A	0.6986	0.8798	0.5953	0.038*	
H31B	0.7541	0.7832	0.6343	0.038*	
H31C	0.8041	0.8852	0.6660	0.038*	
C32	0.46006 (14)	0.84029 (11)	0.58214 (8)	0.0258 (3)	
H32A	0.5154	0.8217	0.5487	0.031*	
H32B	0.3792	0.8093	0.5649	0.031*	
C33	0.44268 (16)	0.94792 (13)	0.57844 (9)	0.0359 (4)	
H33A	0.4017	0.9662	0.5297	0.054*	
H33B	0.5233	0.9795	0.5899	0.054*	
H33C	0.3918	0.9678	0.6133	0.054*	

### Tetraethylazanium cyanodiphenyl-λ4-boranyl diphenylborinate (3) Atomic displacement parameters (Å^2^)

**Table:**

	*U*^11^	*U*^22^	*U*^33^	*U*^12^	*U*^13^	*U*^23^
O1	0.0185 (5)	0.0189 (5)	0.0140 (4)	0.0017 (4)	0.0045 (4)	0.0036 (4)
N1	0.0202 (6)	0.0224 (6)	0.0246 (6)	−0.0005 (5)	0.0043 (5)	−0.0017 (5)
B1	0.0171 (7)	0.0156 (7)	0.0127 (6)	−0.0001 (5)	0.0034 (5)	0.0017 (5)
B2	0.0143 (7)	0.0180 (7)	0.0163 (7)	−0.0005 (5)	0.0008 (5)	0.0015 (6)
C1	0.0137 (6)	0.0214 (7)	0.0141 (6)	0.0027 (5)	0.0028 (5)	0.0022 (5)
C2	0.0160 (6)	0.0164 (6)	0.0137 (6)	−0.0023 (5)	0.0018 (5)	−0.0023 (5)
C3	0.0200 (6)	0.0169 (6)	0.0129 (6)	−0.0007 (5)	0.0019 (5)	0.0006 (5)
C4	0.0211 (7)	0.0241 (7)	0.0136 (6)	−0.0030 (5)	0.0055 (5)	−0.0008 (5)
C5	0.0235 (7)	0.0225 (7)	0.0249 (7)	−0.0005 (6)	0.0100 (6)	−0.0067 (6)
C6	0.0317 (8)	0.0158 (7)	0.0369 (9)	0.0025 (6)	0.0147 (7)	0.0002 (6)
C7	0.0264 (7)	0.0179 (7)	0.0275 (7)	−0.0012 (6)	0.0127 (6)	0.0033 (6)
C8	0.0168 (6)	0.0175 (6)	0.0131 (6)	0.0010 (5)	0.0051 (5)	−0.0010 (5)
C9	0.0193 (6)	0.0178 (6)	0.0166 (6)	0.0001 (5)	0.0043 (5)	−0.0008 (5)
C10	0.0251 (7)	0.0209 (7)	0.0166 (6)	0.0060 (6)	0.0028 (5)	0.0007 (5)
C11	0.0184 (7)	0.0359 (8)	0.0174 (7)	0.0031 (6)	−0.0001 (5)	−0.0033 (6)
C12	0.0222 (7)	0.0361 (9)	0.0241 (7)	−0.0107 (6)	0.0011 (6)	0.0019 (6)
C13	0.0248 (7)	0.0258 (8)	0.0188 (7)	−0.0057 (6)	0.0015 (6)	0.0054 (6)
C14	0.0168 (6)	0.0178 (6)	0.0199 (7)	−0.0023 (5)	0.0057 (5)	0.0027 (5)
C15	0.0199 (7)	0.0241 (7)	0.0187 (7)	0.0003 (5)	0.0070 (5)	0.0022 (6)
C16	0.0223 (7)	0.0220 (7)	0.0268 (7)	0.0031 (6)	0.0068 (6)	0.0099 (6)
C17	0.0253 (7)	0.0163 (7)	0.0336 (8)	0.0010 (6)	0.0135 (6)	−0.0009 (6)
C18	0.0263 (7)	0.0227 (7)	0.0199 (7)	−0.0033 (6)	0.0058 (6)	−0.0024 (6)
C19	0.0213 (7)	0.0183 (7)	0.0190 (7)	−0.0014 (5)	0.0038 (5)	0.0011 (5)
C20	0.0173 (6)	0.0165 (6)	0.0134 (6)	0.0008 (5)	0.0007 (5)	−0.0013 (5)
C21	0.0162 (6)	0.0171 (6)	0.0171 (6)	0.0019 (5)	0.0004 (5)	−0.0009 (5)
C22	0.0204 (7)	0.0241 (7)	0.0156 (6)	−0.0021 (5)	0.0041 (5)	−0.0007 (5)
C23	0.0274 (7)	0.0159 (7)	0.0171 (6)	−0.0011 (5)	0.0018 (5)	0.0023 (5)
C24	0.0269 (7)	0.0167 (7)	0.0191 (7)	0.0041 (5)	0.0035 (6)	−0.0008 (5)
C25	0.0221 (7)	0.0205 (7)	0.0139 (6)	0.0025 (5)	0.0046 (5)	0.0000 (5)
N2	0.0149 (5)	0.0198 (6)	0.0150 (5)	−0.0009 (4)	0.0027 (4)	−0.0014 (4)
C26	0.0193 (7)	0.0205 (7)	0.0289 (8)	0.0013 (5)	0.0050 (6)	−0.0069 (6)
C27	0.0234 (7)	0.0226 (7)	0.0292 (8)	−0.0040 (6)	0.0031 (6)	−0.0065 (6)
C28	0.0192 (7)	0.0221 (7)	0.0220 (7)	0.0015 (5)	0.0081 (5)	−0.0015 (6)
C29	0.0309 (8)	0.0265 (8)	0.0197 (7)	−0.0026 (6)	0.0089 (6)	−0.0002 (6)
C30	0.0180 (6)	0.0224 (7)	0.0189 (7)	−0.0031 (5)	0.0020 (5)	−0.0033 (5)
C31	0.0185 (7)	0.0330 (8)	0.0259 (7)	−0.0043 (6)	0.0056 (6)	−0.0045 (6)
C32	0.0216 (7)	0.0379 (9)	0.0171 (7)	−0.0031 (6)	0.0014 (5)	0.0040 (6)
C33	0.0328 (9)	0.0409 (10)	0.0355 (9)	0.0058 (7)	0.0102 (7)	0.0193 (8)

### Tetraethylazanium cyanodiphenyl-λ4-boranyl diphenylborinate (3) Geometric parameters (Å, º)

**Table:**

O1—B2	1.3350 (17)	C14—C15	1.4036 (19)
O1—B1	1.4831 (16)	C14—C19	1.4059 (19)
N1—C1	1.1518 (18)	C15—C16	1.387 (2)
B1—C8	1.6293 (19)	C16—C17	1.383 (2)
B1—C2	1.6343 (19)	C17—C18	1.385 (2)
B1—C1	1.636 (2)	C18—C19	1.388 (2)
B2—C20	1.591 (2)	C20—C21	1.4045 (18)
B2—C14	1.596 (2)	C20—C25	1.4074 (18)
C2—C3	1.4019 (18)	C21—C22	1.3923 (19)
C2—C7	1.4021 (19)	C22—C23	1.386 (2)
C3—C4	1.3923 (19)	C23—C24	1.385 (2)
C4—C5	1.389 (2)	C24—C25	1.3869 (19)
C5—C6	1.386 (2)	N2—C26	1.5184 (18)
C6—C7	1.395 (2)	N2—C30	1.5202 (17)
C8—C13	1.4004 (19)	N2—C28	1.5239 (17)
C8—C9	1.4001 (18)	N2—C32	1.5249 (18)
C9—C10	1.3940 (19)	C26—C27	1.5158 (19)
C10—C11	1.385 (2)	C28—C29	1.515 (2)
C11—C12	1.386 (2)	C30—C31	1.5185 (19)
C12—C13	1.394 (2)	C32—C33	1.512 (2)
			
B2—O1—B1	138.29 (11)	C15—C14—C19	116.60 (13)
O1—B1—C8	110.58 (11)	C15—C14—B2	122.99 (12)
O1—B1—C2	108.69 (10)	C19—C14—B2	120.41 (12)
C8—B1—C2	111.74 (10)	C16—C15—C14	121.56 (13)
O1—B1—C1	110.69 (10)	C17—C16—C15	120.41 (13)
C8—B1—C1	108.42 (10)	C16—C17—C18	119.56 (13)
C2—B1—C1	106.67 (10)	C17—C18—C19	119.93 (13)
O1—B2—C20	116.03 (12)	C18—C19—C14	121.90 (13)
O1—B2—C14	125.05 (12)	C21—C20—C25	116.67 (12)
C20—B2—C14	118.91 (11)	C21—C20—B2	123.39 (12)
C3—C2—C7	116.17 (12)	C25—C20—B2	119.92 (12)
C3—C2—B1	122.11 (11)	C22—C21—C20	122.06 (12)
C7—C2—B1	121.72 (12)	C23—C22—C21	119.52 (13)
C4—C3—C2	122.36 (12)	C24—C23—C22	119.93 (13)
C5—C4—C3	119.86 (13)	C23—C24—C25	120.30 (13)
C6—C5—C4	119.42 (13)	C24—C25—C20	121.50 (13)
C5—C6—C7	120.09 (14)	C26—N2—C30	108.41 (10)
N1—C1—B1	177.10 (13)	C26—N2—C28	111.41 (10)
C6—C7—C2	122.08 (13)	C30—N2—C28	108.52 (10)
C13—C8—C9	116.23 (12)	C26—N2—C32	108.87 (11)
C13—C8—B1	120.44 (12)	C30—N2—C32	111.12 (10)
C9—C8—B1	123.31 (12)	C28—N2—C32	108.52 (10)
C10—C9—C8	122.12 (13)	C27—C26—N2	114.71 (11)
C11—C10—C9	120.21 (13)	C29—C28—N2	115.19 (11)
C10—C11—C12	119.08 (13)	C31—C30—N2	114.89 (11)
C11—C12—C13	120.29 (14)	C33—C32—N2	114.87 (13)
C12—C13—C8	122.02 (13)		

### Bis[(2.2.2-cryptand)potassium] 2,2,4,6-tetraphenyl-1,3,5,2λ4,4,6-trioxatriborinan-2-ide cyanomethyldiphenylborate tetrahydrofuran disolvate (4) Crystal data

**Table:**

2C_18_H_36_KN_2_O_6_^+^·C_24_H_20_B_3_O_3_^−^·C_14_H_13_BN^−^·2C_4_H_8_O	*D*_x_ = 1.242 Mg m^−^^3^
*M**_r_* = 1570.27	Mo *K*α radiation, λ = 0.71073 Å
Orthorhombic, *P**c**a*2_1_	Cell parameters from 9334 reflections
*a* = 27.193 (2) Å	θ = 2.3–25.3°
*b* = 14.5520 (11) Å	µ = 0.18 mm^−^^1^
*c* = 21.2218 (16) Å	*T* = 88 K
*V* = 8397.7 (11) Å^3^	Irregular, colorless
*Z* = 4	0.45 × 0.33 × 0.28 mm
*F*(000) = 3368	

### Bis[(2.2.2-cryptand)potassium] 2,2,4,6-tetraphenyl-1,3,5,2λ4,4,6-trioxatriborinan-2-ide cyanomethyldiphenylborate tetrahydrofuran disolvate (4) Data collection

**Table:**

Bruker SMART APEXII CCD diffractometer	14236 reflections with *I* > 2σ(*I*)
Radiation source: fine-focus sealed tube	*R*_int_ = 0.038
φ and ω scans	θ_max_ = 25.7°, θ_min_ = 1.6°
Absorption correction: multi-scan (SADABS; Krause et al., 2015)	*h* = −33→33
*T*_min_ = 0.825, *T*_max_ = 0.862	*k* = −17→17
85621 measured reflections	*l* = −25→25
15958 independent reflections	

### Bis[(2.2.2-cryptand)potassium] 2,2,4,6-tetraphenyl-1,3,5,2λ4,4,6-trioxatriborinan-2-ide cyanomethyldiphenylborate tetrahydrofuran disolvate (4) Refinement

**Table:**

Refinement on *F*^2^	Primary atom site location: dual space
Least-squares matrix: full	Secondary atom site location: difference Fourier map
*R*[*F*^2^ > 2σ(*F*^2^)] = 0.074	Hydrogen site location: inferred from neighbouring sites
*wR*(*F*^2^) = 0.209	H-atom parameters constrained
*S* = 1.06	*w* = 1/[σ^2^(*F*_o_^2^) + (0.1366*P*)^2^ + 5.6525*P*] where *P* = (*F*_o_^2^ + 2*F*_c_^2^)/3
15958 reflections	(Δ/σ)_max_ = 0.001
988 parameters	Δρ_max_ = 0.78 e Å^−^^3^
1 restraint	Δρ_min_ = −0.34 e Å^−^^3^

### Bis[(2.2.2-cryptand)potassium] 2,2,4,6-tetraphenyl-1,3,5,2λ4,4,6-trioxatriborinan-2-ide cyanomethyldiphenylborate tetrahydrofuran disolvate (4) Special details

**Table:**

Geometry. All esds (except the esd in the dihedral angle between two l.s. planes) are estimated using the full covariance matrix. The cell esds are taken into account individually in the estimation of esds in distances, angles and torsion angles; correlations between esds in cell parameters are only used when they are defined by crystal symmetry. An approximate (isotropic) treatment of cell esds is used for estimating esds involving l.s. planes.
Refinement. A colorless crystal of approximate dimensions 0.280 x 0.325 x 0.454 mm was mounted in a cryoloop and transferred to a Bruker SMART APEX II diffractometer. The APEX2 program package was used to determine the unit-cell parameters and for data collection (60 sec/frame scan time for a sphere of diffraction data). The raw frame data was processed using SAINT and SADABS to yield the reflection data file. Subsequent calculations were carried out using the SHELXTL program. The diffraction symmetry was mmm and the systematic absences were consistent with the orthorhombic space groups Pbcm and Pca21. It was later determined that space group Pca21 was correct.The structure was solved by dual space methods and refined on F2 by full-matrix least-squares techniques. The analytical scattering factors for neutral atoms were used throughout the analysis. Hydrogen atoms were included using a riding model. There were two molecules of tetrahydrofuran solvent present. One solvent molecule was disordered and included using multiple components with partial site-occupancy-factors.Least-squares analysis yielded wR2 = 0.2089 and Goof = 1.064 for 988 variables refined against 15958 data (0.82 Å), R1 = 0.0742 for those 14236 data with I > 2.0sigma(I). The structure was refined as a two component inversion twin.

### Bis[(2.2.2-cryptand)potassium] 2,2,4,6-tetraphenyl-1,3,5,2λ4,4,6-trioxatriborinan-2-ide cyanomethyldiphenylborate tetrahydrofuran disolvate (4) Fractional atomic coordinates and isotropic or equivalent isotropic displacement parameters (Å^2^)

**Table:**

	*x*	*y*	*z*	*U*_iso_*/*U*_eq_	Occ. (<1)
B1	0.8783 (3)	0.5804 (5)	0.3189 (4)	0.0416 (15)	
B2	0.7983 (2)	0.5517 (5)	0.3727 (3)	0.0363 (14)	
B3	0.8194 (2)	0.7051 (5)	0.3472 (3)	0.0357 (14)	
O1	0.84187 (16)	0.5214 (3)	0.3531 (2)	0.0438 (10)	
O2	0.78518 (15)	0.6428 (3)	0.3691 (2)	0.0409 (10)	
O3	0.86284 (16)	0.6798 (3)	0.3227 (2)	0.0439 (10)	
C1	0.9323 (2)	0.5724 (4)	0.3521 (3)	0.0373 (13)	
C2	0.9683 (2)	0.6407 (4)	0.3493 (3)	0.0358 (13)	
H2A	0.9617	0.6944	0.3254	0.043*	
C3	1.0129 (2)	0.6337 (4)	0.3795 (3)	0.0371 (13)	
H3A	1.0363	0.6819	0.3765	0.044*	
C4	1.0229 (3)	0.5565 (6)	0.4139 (4)	0.059 (2)	
H4A	1.0536	0.5503	0.4348	0.071*	
C5	0.9875 (3)	0.4864 (6)	0.4181 (4)	0.069 (2)	
H5A	0.9938	0.4331	0.4427	0.083*	
C6	0.9438 (3)	0.4954 (5)	0.3866 (4)	0.0526 (17)	
H6A	0.9205	0.4467	0.3887	0.063*	
C7	0.8806 (2)	0.5476 (4)	0.2443 (3)	0.0407 (14)	
C8	0.8981 (2)	0.6029 (6)	0.1968 (4)	0.0556 (19)	
H8A	0.9101	0.6625	0.2067	0.067*	
C9	0.8986 (3)	0.5735 (7)	0.1358 (4)	0.071 (2)	
H9A	0.9106	0.6134	0.1039	0.085*	
C10	0.8822 (3)	0.4880 (7)	0.1195 (3)	0.067 (2)	
H10A	0.8833	0.4685	0.0768	0.081*	
C11	0.8641 (3)	0.4304 (6)	0.1651 (4)	0.060 (2)	
H11A	0.8521	0.3712	0.1541	0.072*	
C12	0.8633 (2)	0.4599 (5)	0.2283 (4)	0.0454 (16)	
H12A	0.8511	0.4202	0.2601	0.054*	
C13	0.7585 (2)	0.4816 (5)	0.3991 (3)	0.0424 (14)	
C14	0.7634 (3)	0.3885 (5)	0.3920 (3)	0.0503 (16)	
H14A	0.7918	0.3640	0.3720	0.060*	
C15	0.7271 (3)	0.3302 (5)	0.4140 (4)	0.0558 (19)	
H15A	0.7321	0.2658	0.4104	0.067*	
C16	0.6842 (3)	0.3609 (7)	0.4406 (4)	0.068 (2)	
H16A	0.6594	0.3187	0.4533	0.082*	
C17	0.6773 (3)	0.4565 (5)	0.4489 (4)	0.0547 (18)	
H17A	0.6482	0.4809	0.4671	0.066*	
C18	0.7150 (2)	0.5120 (5)	0.4290 (3)	0.0455 (15)	
H18A	0.7117	0.5762	0.4359	0.055*	
C19	0.8057 (2)	0.8116 (5)	0.3545 (3)	0.0435 (14)	
C20	0.7603 (3)	0.8361 (4)	0.3792 (3)	0.0457 (15)	
H20A	0.7384	0.7892	0.3928	0.055*	
C21	0.7462 (3)	0.9267 (5)	0.3846 (4)	0.0552 (17)	
H21A	0.7146	0.9416	0.4008	0.066*	
C22	0.7791 (2)	0.9987 (5)	0.3657 (3)	0.0451 (15)	
H22A	0.7707	1.0617	0.3702	0.054*	
C23	0.8234 (3)	0.9716 (4)	0.3409 (3)	0.0442 (15)	
H23A	0.8455	1.0174	0.3264	0.053*	
C24	0.8372 (2)	0.8797 (4)	0.3362 (3)	0.0411 (14)	
H24A	0.8688	0.8642	0.3201	0.049*	
B4	0.6001 (3)	0.1179 (6)	0.5064 (5)	0.054 (2)	
N1	0.6889 (3)	0.1126 (5)	0.4428 (3)	0.0676 (19)	
C25	0.6501 (2)	0.1121 (5)	0.4657 (4)	0.0526 (19)	
C26	0.5909 (2)	0.2136 (4)	0.5281 (3)	0.0405 (14)	
H26A	0.6005	0.2195	0.5724	0.061*	
H26B	0.5558	0.2279	0.5237	0.061*	
H26C	0.6102	0.2564	0.5025	0.061*	
C27	0.6088 (2)	0.0457 (5)	0.5642 (3)	0.0436 (15)	
C28	0.5940 (2)	0.0708 (6)	0.6250 (4)	0.062 (2)	
H28A	0.5778	0.1279	0.6315	0.074*	
C29	0.6028 (3)	0.0116 (8)	0.6771 (4)	0.074 (3)	
H29A	0.5939	0.0302	0.7185	0.089*	
C30	0.6238 (3)	−0.0709 (8)	0.6675 (5)	0.070 (3)	
H30A	0.6291	−0.1110	0.7022	0.084*	
C31	0.6373 (3)	−0.0972 (6)	0.6097 (4)	0.062 (2)	
H31A	0.6521	−0.1557	0.6036	0.074*	
C32	0.6300 (2)	−0.0405 (5)	0.5593 (4)	0.0457 (16)	
H32A	0.6400	−0.0612	0.5188	0.055*	
C33	0.5551 (2)	0.0858 (4)	0.4593 (3)	0.0444 (15)	
C34	0.5151 (2)	0.1423 (4)	0.4492 (3)	0.0413 (14)	
H34A	0.5147	0.2013	0.4683	0.050*	
C35	0.4758 (2)	0.1170 (5)	0.4127 (3)	0.0423 (15)	
H35A	0.4493	0.1587	0.4074	0.051*	
C36	0.4739 (2)	0.0349 (5)	0.3842 (4)	0.0493 (16)	
H36A	0.4462	0.0179	0.3595	0.059*	
C37	0.5126 (3)	−0.0236 (6)	0.3918 (5)	0.074 (3)	
H37A	0.5117	−0.0827	0.3728	0.089*	
C38	0.5538 (3)	0.0023 (6)	0.4272 (5)	0.067 (2)	
H38A	0.5813	−0.0378	0.4295	0.081*	
K1	0.63521 (4)	0.17698 (7)	0.13192 (5)	0.0225 (2)	
O4	0.6908 (2)	0.1969 (3)	0.2438 (2)	0.0539 (13)	
O5	0.59021 (16)	0.1512 (3)	0.2501 (2)	0.0440 (10)	
O6	0.68052 (14)	0.3229 (2)	0.0665 (2)	0.0321 (9)	
O7	0.57967 (15)	0.3352 (3)	0.0934 (2)	0.0370 (9)	
O8	0.68719 (13)	0.0226 (3)	0.0800 (2)	0.0323 (9)	
O9	0.58499 (13)	0.0382 (3)	0.06660 (19)	0.0316 (8)	
N2	0.74407 (17)	0.1841 (3)	0.1252 (3)	0.0399 (12)	
N3	0.52603 (17)	0.1712 (3)	0.1390 (3)	0.0367 (11)	
C39	0.7649 (3)	0.2152 (5)	0.1857 (4)	0.0568 (19)	
H39A	0.7612	0.2827	0.1886	0.068*	
H39B	0.8006	0.2013	0.1860	0.068*	
C40	0.7421 (3)	0.1730 (5)	0.2421 (4)	0.065 (2)	
H40A	0.7459	0.1053	0.2404	0.077*	
H40B	0.7587	0.1955	0.2806	0.077*	
C41	0.6699 (3)	0.1606 (5)	0.2991 (3)	0.0543 (19)	
H41A	0.6882	0.1831	0.3364	0.065*	
H41B	0.6719	0.0927	0.2984	0.065*	
C42	0.6184 (3)	0.1899 (5)	0.3028 (3)	0.0520 (18)	
H42A	0.6166	0.2578	0.3014	0.062*	
H42B	0.6040	0.1690	0.3431	0.062*	
C43	0.5393 (3)	0.1761 (5)	0.2556 (3)	0.0495 (17)	
H43A	0.5358	0.2438	0.2549	0.059*	
H43B	0.5258	0.1531	0.2960	0.059*	
C44	0.5117 (2)	0.1341 (5)	0.2009 (3)	0.0485 (16)	
H44A	0.5173	0.0669	0.2009	0.058*	
H44B	0.4760	0.1445	0.2071	0.058*	
C45	0.7583 (2)	0.2471 (4)	0.0750 (3)	0.0430 (15)	
H45A	0.7518	0.2173	0.0339	0.052*	
H45B	0.7941	0.2585	0.0779	0.052*	
C46	0.7319 (2)	0.3381 (4)	0.0766 (3)	0.0406 (15)	
H46A	0.7371	0.3682	0.1179	0.049*	
H46B	0.7451	0.3790	0.0434	0.049*	
C47	0.6549 (2)	0.4078 (4)	0.0635 (3)	0.0367 (13)	
H47A	0.6704	0.4481	0.0316	0.044*	
H47B	0.6566	0.4391	0.1048	0.044*	
C48	0.6024 (2)	0.3903 (4)	0.0461 (3)	0.0404 (14)	
H48A	0.5847	0.4494	0.0422	0.049*	
H48B	0.6008	0.3583	0.0050	0.049*	
C49	0.5290 (2)	0.3193 (5)	0.0787 (4)	0.0483 (16)	
H49A	0.5263	0.2853	0.0384	0.058*	
H49B	0.5117	0.3787	0.0740	0.058*	
C50	0.5058 (2)	0.2641 (5)	0.1312 (4)	0.0495 (16)	
H50A	0.5100	0.2980	0.1712	0.059*	
H50B	0.4701	0.2590	0.1229	0.059*	
C51	0.7628 (2)	0.0919 (4)	0.1118 (4)	0.0478 (17)	
H51A	0.7610	0.0549	0.1508	0.057*	
H51B	0.7979	0.0969	0.0998	0.057*	
C52	0.7361 (2)	0.0415 (4)	0.0605 (4)	0.0450 (16)	
H52A	0.7356	0.0794	0.0218	0.054*	
H52B	0.7534	−0.0167	0.0510	0.054*	
C53	0.6610 (2)	−0.0244 (4)	0.0311 (3)	0.0382 (13)	
H53A	0.6783	−0.0821	0.0201	0.046*	
H53B	0.6596	0.0146	−0.0071	0.046*	
C54	0.6099 (2)	−0.0454 (4)	0.0535 (3)	0.0356 (13)	
H54A	0.5919	−0.0802	0.0207	0.043*	
H54B	0.6114	−0.0837	0.0920	0.043*	
C55	0.5348 (2)	0.0197 (4)	0.0827 (3)	0.0399 (14)	
H55A	0.5332	−0.0146	0.1230	0.048*	
H55B	0.5192	−0.0182	0.0495	0.048*	
C56	0.5077 (2)	0.1108 (4)	0.0891 (3)	0.0388 (13)	
H56A	0.5098	0.1439	0.0485	0.047*	
H56B	0.4725	0.0978	0.0972	0.047*	
K2	0.37144 (4)	0.32115 (7)	0.67152 (5)	0.0278 (3)	
O10	0.4324 (2)	0.3508 (4)	0.5644 (2)	0.0635 (15)	
O11	0.33319 (18)	0.2979 (4)	0.5500 (2)	0.0503 (12)	
O12	0.42078 (19)	0.1605 (3)	0.7159 (3)	0.0540 (12)	
O13	0.31818 (18)	0.1755 (3)	0.7270 (2)	0.0441 (11)	
O14	0.41652 (16)	0.4584 (3)	0.7459 (2)	0.0422 (10)	
O15	0.31684 (15)	0.4755 (3)	0.7123 (2)	0.0370 (9)	
N4	0.4804 (2)	0.3234 (4)	0.6868 (4)	0.0608 (19)	
N5	0.26389 (19)	0.3164 (3)	0.6549 (3)	0.0411 (13)	
C57	0.5027 (3)	0.3645 (8)	0.6302 (5)	0.088 (3)	
H57A	0.5386	0.3531	0.6311	0.106*	
H57B	0.4976	0.4318	0.6313	0.106*	
C58	0.4825 (4)	0.3279 (10)	0.5704 (5)	0.099 (4)	
H58A	0.5011	0.3538	0.5344	0.119*	
H58B	0.4863	0.2603	0.5695	0.119*	
C59	0.4153 (3)	0.3079 (5)	0.5103 (3)	0.059 (2)	
H59A	0.4170	0.2404	0.5153	0.071*	
H59B	0.4358	0.3256	0.4737	0.071*	
C60	0.3647 (3)	0.3362 (5)	0.4999 (3)	0.057 (2)	
H60A	0.3534	0.3139	0.4583	0.068*	
H60B	0.3626	0.4041	0.5001	0.068*	
C61	0.2829 (3)	0.3242 (5)	0.5402 (4)	0.0575 (19)	
H61A	0.2715	0.3015	0.4987	0.069*	
H61B	0.2801	0.3920	0.5403	0.069*	
C62	0.2513 (3)	0.2849 (5)	0.5910 (4)	0.0525 (17)	
H62A	0.2166	0.3011	0.5823	0.063*	
H62B	0.2539	0.2170	0.5897	0.063*	
C63	0.4985 (3)	0.2297 (6)	0.6946 (5)	0.068 (2)	
H63A	0.5330	0.2324	0.7097	0.081*	
H63B	0.4987	0.1991	0.6530	0.081*	
C64	0.4697 (3)	0.1739 (6)	0.7386 (5)	0.073 (3)	
H64A	0.4859	0.1135	0.7441	0.088*	
H64B	0.4686	0.2047	0.7801	0.088*	
C65	0.3931 (3)	0.1040 (5)	0.7584 (4)	0.062 (2)	
H65A	0.3911	0.1339	0.8002	0.074*	
H65B	0.4095	0.0438	0.7635	0.074*	
C66	0.3428 (3)	0.0905 (4)	0.7324 (4)	0.0543 (19)	
H66A	0.3451	0.0610	0.6905	0.065*	
H66B	0.3240	0.0492	0.7605	0.065*	
C67	0.2685 (3)	0.1627 (4)	0.7081 (4)	0.0492 (18)	
H67A	0.2513	0.1233	0.7391	0.059*	
H67B	0.2675	0.1317	0.6666	0.059*	
C68	0.2434 (3)	0.2546 (4)	0.7039 (4)	0.0523 (18)	
H68A	0.2080	0.2446	0.6950	0.063*	
H68B	0.2459	0.2856	0.7453	0.063*	
C69	0.4936 (3)	0.3785 (5)	0.7419 (5)	0.065 (2)	
H69A	0.5296	0.3887	0.7420	0.078*	
H69B	0.4850	0.3440	0.7806	0.078*	
C70	0.4678 (3)	0.4699 (5)	0.7429 (5)	0.071 (3)	
H70A	0.4791	0.5057	0.7799	0.085*	
H70B	0.4764	0.5049	0.7045	0.085*	
C71	0.3906 (2)	0.5435 (4)	0.7514 (3)	0.0429 (15)	
H71A	0.3940	0.5790	0.7119	0.051*	
H71B	0.4047	0.5803	0.7862	0.051*	
C72	0.3381 (2)	0.5254 (4)	0.7641 (3)	0.0410 (14)	
H72A	0.3347	0.4892	0.8033	0.049*	
H72B	0.3205	0.5843	0.7698	0.049*	
C73	0.2657 (2)	0.4600 (4)	0.7209 (4)	0.0483 (17)	
H73A	0.2487	0.5194	0.7270	0.058*	
H73B	0.2604	0.4221	0.7591	0.058*	
C74	0.2446 (2)	0.4112 (4)	0.6642 (4)	0.0506 (17)	
H74A	0.2084	0.4082	0.6687	0.061*	
H74B	0.2519	0.4480	0.6261	0.061*	
O16	0.8367 (2)	0.2092 (4)	0.3526 (3)	0.0673 (15)	
C75	0.8791 (4)	0.2395 (8)	0.3223 (6)	0.091 (3)	
H75A	0.8781	0.3072	0.3176	0.110*	
H75B	0.8812	0.2119	0.2797	0.110*	
C76	0.9223 (3)	0.2129 (9)	0.3595 (7)	0.103 (4)	
H76A	0.9443	0.2662	0.3664	0.123*	
H76B	0.9410	0.1637	0.3381	0.123*	
C77	0.9007 (4)	0.1783 (6)	0.4226 (6)	0.080 (3)	
H77A	0.9038	0.1107	0.4263	0.096*	
H77B	0.9172	0.2076	0.4589	0.096*	
C78	0.8485 (3)	0.2063 (6)	0.4185 (4)	0.0595 (19)	
H78A	0.8273	0.1615	0.4406	0.071*	
H78B	0.8438	0.2676	0.4379	0.071*	
O17	0.4260 (5)	0.2513 (10)	0.8786 (7)	0.075 (4)*	0.5
C79	0.3802 (9)	0.3057 (16)	0.8880 (10)	0.072 (5)*	0.5
H79A	0.3533	0.2838	0.8604	0.087*	0.5
H79B	0.3859	0.3720	0.8802	0.087*	0.5
C80	0.3684 (7)	0.2852 (15)	0.9628 (9)	0.068 (5)*	0.5
H80A	0.3447	0.3291	0.9814	0.081*	0.5
H80B	0.3578	0.2211	0.9708	0.081*	0.5
C81	0.4237 (13)	0.304 (2)	0.9836 (18)	0.123 (11)*	0.5
H81A	0.4314	0.3706	0.9806	0.148*	0.5
H81B	0.4293	0.2839	1.0275	0.148*	0.5
C82	0.4539 (10)	0.2522 (19)	0.9406 (14)	0.103 (7)*	0.5
H82A	0.4588	0.1889	0.9564	0.123*	0.5
H82B	0.4864	0.2818	0.9353	0.123*	0.5
O17B	0.4414 (7)	0.2715 (13)	0.9034 (10)	0.100 (5)*	0.5
C79B	0.4032 (9)	0.3234 (16)	0.8637 (13)	0.089 (6)*	0.5
H79C	0.4046	0.3904	0.8718	0.106*	0.5
H79D	0.4081	0.3121	0.8181	0.106*	0.5
C80B	0.3583 (8)	0.2855 (14)	0.8849 (9)	0.066 (5)*	0.5
H80C	0.3326	0.3336	0.8838	0.079*	0.5
H80D	0.3482	0.2359	0.8557	0.079*	0.5
C81B	0.3612 (7)	0.2500 (15)	0.9457 (10)	0.070 (5)*	0.5
H81C	0.3536	0.1835	0.9449	0.084*	0.5
H81D	0.3365	0.2807	0.9729	0.084*	0.5
C82B	0.4160 (8)	0.2659 (16)	0.9746 (11)	0.074 (5)*	0.5
H82C	0.4279	0.2133	0.9998	0.089*	0.5
H82D	0.4190	0.3239	0.9987	0.089*	0.5

### Bis[(2.2.2-cryptand)potassium] 2,2,4,6-tetraphenyl-1,3,5,2λ4,4,6-trioxatriborinan-2-ide cyanomethyldiphenylborate tetrahydrofuran disolvate (4) Atomic displacement parameters (Å^2^)

**Table:**

	*U*^11^	*U*^22^	*U*^33^	*U*^12^	*U*^13^	*U*^23^
B1	0.043 (4)	0.041 (4)	0.040 (4)	0.000 (3)	0.008 (3)	0.000 (3)
B2	0.035 (3)	0.041 (4)	0.034 (3)	0.000 (3)	−0.001 (3)	0.000 (3)
B3	0.036 (3)	0.037 (3)	0.034 (3)	−0.001 (3)	0.001 (3)	0.005 (3)
O1	0.041 (2)	0.038 (2)	0.052 (3)	−0.0030 (18)	0.007 (2)	0.0022 (19)
O2	0.039 (2)	0.037 (2)	0.046 (2)	−0.0065 (18)	0.0003 (19)	0.0097 (19)
O3	0.045 (2)	0.038 (2)	0.049 (3)	−0.0006 (18)	0.007 (2)	0.0067 (19)
C1	0.040 (3)	0.036 (3)	0.036 (3)	0.005 (2)	0.003 (2)	−0.010 (3)
C2	0.049 (3)	0.031 (3)	0.028 (3)	0.005 (2)	0.010 (2)	−0.008 (2)
C3	0.042 (3)	0.034 (3)	0.035 (3)	−0.005 (2)	0.017 (3)	−0.010 (2)
C4	0.037 (4)	0.087 (6)	0.053 (4)	0.006 (4)	−0.007 (3)	−0.005 (4)
C5	0.067 (5)	0.061 (5)	0.080 (6)	−0.002 (4)	−0.009 (4)	0.036 (4)
C6	0.044 (4)	0.042 (3)	0.072 (5)	−0.007 (3)	0.002 (3)	0.015 (3)
C7	0.031 (3)	0.048 (4)	0.043 (3)	0.005 (3)	0.001 (3)	0.001 (3)
C8	0.034 (3)	0.079 (5)	0.054 (4)	−0.019 (3)	−0.005 (3)	0.012 (4)
C9	0.041 (4)	0.115 (8)	0.056 (5)	−0.019 (4)	0.005 (3)	0.021 (5)
C10	0.045 (4)	0.124 (8)	0.033 (4)	0.009 (5)	−0.013 (3)	−0.021 (4)
C11	0.046 (4)	0.066 (5)	0.069 (5)	0.004 (3)	−0.017 (4)	−0.019 (4)
C12	0.037 (3)	0.043 (4)	0.055 (4)	0.008 (3)	0.005 (3)	0.001 (3)
C13	0.045 (3)	0.055 (4)	0.028 (3)	−0.003 (3)	−0.006 (2)	0.002 (3)
C14	0.056 (4)	0.045 (4)	0.049 (4)	−0.014 (3)	−0.008 (3)	0.006 (3)
C15	0.074 (5)	0.037 (3)	0.057 (4)	−0.018 (3)	−0.002 (4)	0.009 (3)
C16	0.056 (5)	0.095 (7)	0.053 (4)	−0.030 (4)	−0.010 (4)	0.022 (4)
C17	0.040 (3)	0.063 (5)	0.061 (4)	−0.008 (3)	−0.007 (3)	0.026 (4)
C18	0.033 (3)	0.062 (4)	0.041 (3)	−0.003 (3)	0.000 (3)	0.003 (3)
C19	0.043 (3)	0.053 (4)	0.034 (3)	−0.001 (3)	−0.005 (3)	0.004 (3)
C20	0.049 (4)	0.039 (3)	0.049 (4)	−0.001 (3)	0.010 (3)	0.005 (3)
C21	0.052 (4)	0.057 (4)	0.056 (4)	0.014 (3)	−0.006 (3)	−0.013 (4)
C22	0.048 (4)	0.041 (3)	0.047 (4)	0.005 (3)	−0.010 (3)	0.007 (3)
C23	0.058 (4)	0.034 (3)	0.041 (3)	−0.004 (3)	−0.011 (3)	−0.001 (3)
C24	0.050 (4)	0.036 (3)	0.037 (3)	−0.002 (3)	−0.009 (3)	0.001 (3)
B4	0.041 (4)	0.044 (4)	0.076 (6)	−0.004 (3)	−0.001 (4)	0.013 (4)
N1	0.086 (5)	0.062 (4)	0.055 (4)	0.019 (4)	−0.008 (4)	0.002 (3)
C25	0.023 (3)	0.063 (4)	0.071 (5)	−0.004 (3)	0.015 (3)	0.032 (4)
C26	0.039 (3)	0.020 (3)	0.062 (4)	0.001 (2)	−0.012 (3)	−0.005 (3)
C27	0.028 (3)	0.049 (4)	0.054 (4)	−0.006 (3)	−0.003 (3)	0.007 (3)
C28	0.025 (3)	0.068 (5)	0.092 (6)	0.002 (3)	−0.001 (3)	−0.032 (5)
C29	0.039 (4)	0.144 (9)	0.038 (4)	−0.019 (5)	0.002 (3)	−0.013 (5)
C30	0.033 (4)	0.105 (7)	0.073 (6)	−0.016 (4)	−0.014 (4)	0.027 (6)
C31	0.040 (4)	0.065 (5)	0.080 (6)	−0.006 (3)	−0.008 (4)	0.030 (4)
C32	0.036 (3)	0.042 (4)	0.060 (4)	−0.006 (3)	0.007 (3)	0.005 (3)
C33	0.039 (3)	0.041 (3)	0.053 (4)	−0.009 (3)	0.008 (3)	0.011 (3)
C34	0.052 (4)	0.032 (3)	0.040 (3)	−0.001 (3)	0.008 (3)	0.010 (3)
C35	0.042 (3)	0.048 (4)	0.036 (3)	0.008 (3)	0.011 (3)	0.016 (3)
C36	0.035 (3)	0.048 (4)	0.065 (4)	−0.001 (3)	−0.002 (3)	0.002 (3)
C37	0.053 (5)	0.051 (4)	0.118 (8)	0.013 (3)	−0.023 (5)	−0.037 (5)
C38	0.033 (4)	0.065 (5)	0.103 (7)	0.012 (3)	−0.008 (4)	−0.006 (5)
K1	0.0211 (5)	0.0246 (5)	0.0218 (5)	−0.0034 (4)	0.0017 (4)	−0.0003 (4)
O4	0.073 (3)	0.059 (3)	0.031 (2)	−0.024 (3)	−0.012 (2)	0.008 (2)
O5	0.044 (2)	0.060 (3)	0.028 (2)	−0.008 (2)	0.0118 (18)	−0.0045 (19)
O6	0.0296 (19)	0.0231 (19)	0.044 (2)	−0.0051 (14)	0.0056 (17)	0.0003 (16)
O7	0.031 (2)	0.034 (2)	0.046 (2)	0.0045 (16)	0.0025 (17)	0.0027 (18)
O8	0.0252 (18)	0.0274 (19)	0.044 (2)	−0.0023 (14)	0.0005 (16)	−0.0018 (17)
O9	0.0266 (18)	0.033 (2)	0.035 (2)	−0.0052 (15)	−0.0020 (15)	−0.0078 (16)
N2	0.024 (2)	0.037 (3)	0.058 (3)	−0.0057 (19)	−0.013 (2)	−0.002 (2)
N3	0.027 (2)	0.037 (2)	0.046 (3)	−0.0024 (18)	0.008 (2)	−0.005 (2)
C39	0.049 (4)	0.052 (4)	0.070 (5)	−0.015 (3)	−0.025 (4)	−0.001 (4)
C40	0.066 (5)	0.062 (5)	0.066 (5)	−0.014 (4)	−0.043 (4)	0.015 (4)
C41	0.090 (6)	0.036 (3)	0.037 (3)	−0.008 (3)	−0.013 (4)	0.004 (3)
C42	0.081 (5)	0.055 (4)	0.020 (3)	0.008 (4)	0.003 (3)	−0.004 (3)
C43	0.050 (4)	0.062 (4)	0.037 (3)	−0.007 (3)	0.024 (3)	−0.005 (3)
C44	0.041 (3)	0.054 (4)	0.050 (4)	−0.018 (3)	0.019 (3)	−0.013 (3)
C45	0.020 (3)	0.044 (3)	0.065 (4)	−0.011 (2)	0.006 (3)	−0.002 (3)
C46	0.036 (3)	0.028 (3)	0.058 (4)	−0.012 (2)	0.013 (3)	0.001 (3)
C47	0.045 (3)	0.021 (3)	0.044 (3)	0.000 (2)	0.013 (3)	0.004 (2)
C48	0.045 (3)	0.030 (3)	0.046 (4)	0.010 (3)	−0.002 (3)	0.011 (3)
C49	0.035 (3)	0.044 (4)	0.067 (5)	0.009 (3)	0.001 (3)	−0.001 (3)
C50	0.027 (3)	0.048 (4)	0.074 (5)	0.006 (2)	0.013 (3)	−0.006 (4)
C51	0.024 (3)	0.038 (3)	0.082 (5)	0.000 (2)	−0.010 (3)	0.000 (3)
C52	0.022 (3)	0.037 (3)	0.076 (5)	0.006 (2)	0.004 (3)	−0.001 (3)
C53	0.039 (3)	0.039 (3)	0.037 (3)	0.004 (3)	0.001 (2)	−0.009 (3)
C54	0.038 (3)	0.030 (3)	0.038 (3)	−0.007 (2)	−0.006 (2)	−0.006 (2)
C55	0.028 (3)	0.040 (3)	0.051 (3)	−0.013 (2)	−0.003 (2)	−0.009 (3)
C56	0.021 (2)	0.052 (4)	0.044 (3)	−0.004 (2)	0.001 (2)	−0.001 (3)
K2	0.0351 (6)	0.0252 (6)	0.0231 (5)	−0.0068 (4)	0.0050 (4)	−0.0041 (4)
O10	0.072 (4)	0.078 (4)	0.041 (3)	−0.021 (3)	0.015 (3)	−0.002 (3)
O11	0.055 (3)	0.067 (3)	0.029 (2)	0.001 (2)	−0.006 (2)	−0.004 (2)
O12	0.058 (3)	0.045 (3)	0.059 (3)	0.008 (2)	0.000 (2)	−0.004 (2)
O13	0.062 (3)	0.024 (2)	0.046 (3)	−0.0058 (18)	0.020 (2)	0.0017 (17)
O14	0.043 (2)	0.031 (2)	0.053 (3)	−0.0010 (18)	−0.005 (2)	−0.0147 (19)
O15	0.033 (2)	0.036 (2)	0.042 (2)	−0.0053 (17)	0.0069 (17)	−0.0058 (18)
N4	0.045 (3)	0.056 (4)	0.081 (5)	−0.009 (3)	0.013 (3)	−0.031 (3)
N5	0.035 (3)	0.032 (3)	0.056 (3)	−0.008 (2)	0.006 (2)	−0.006 (2)
C57	0.055 (5)	0.107 (8)	0.102 (8)	−0.039 (5)	0.032 (5)	−0.022 (7)
C58	0.064 (6)	0.156 (11)	0.077 (7)	−0.011 (6)	0.046 (6)	0.011 (7)
C59	0.087 (6)	0.046 (4)	0.043 (4)	0.006 (4)	0.032 (4)	0.006 (3)
C60	0.089 (6)	0.059 (4)	0.023 (3)	0.009 (4)	0.008 (3)	−0.002 (3)
C61	0.064 (5)	0.051 (4)	0.057 (4)	−0.010 (3)	−0.018 (4)	−0.005 (3)
C62	0.044 (3)	0.042 (3)	0.072 (5)	−0.010 (3)	−0.013 (3)	−0.014 (3)
C63	0.041 (4)	0.075 (5)	0.087 (6)	0.011 (4)	0.006 (4)	−0.030 (5)
C64	0.068 (5)	0.059 (5)	0.093 (7)	0.029 (4)	−0.016 (5)	−0.020 (5)
C65	0.092 (6)	0.036 (4)	0.059 (5)	0.017 (4)	0.006 (4)	0.005 (3)
C66	0.077 (5)	0.030 (3)	0.055 (4)	0.001 (3)	0.018 (4)	0.004 (3)
C67	0.056 (4)	0.036 (3)	0.056 (4)	−0.022 (3)	0.031 (3)	−0.007 (3)
C68	0.043 (4)	0.038 (3)	0.076 (5)	−0.016 (3)	0.023 (3)	−0.005 (3)
C69	0.047 (4)	0.062 (5)	0.086 (6)	0.007 (3)	−0.019 (4)	−0.028 (4)
C70	0.040 (4)	0.052 (4)	0.120 (8)	−0.002 (3)	−0.022 (4)	−0.035 (5)
C71	0.050 (4)	0.033 (3)	0.046 (4)	0.002 (3)	−0.009 (3)	−0.014 (3)
C72	0.050 (4)	0.032 (3)	0.041 (3)	0.003 (3)	0.001 (3)	−0.010 (3)
C73	0.037 (3)	0.030 (3)	0.078 (5)	−0.003 (2)	0.018 (3)	−0.011 (3)
C74	0.030 (3)	0.035 (3)	0.086 (5)	−0.004 (2)	0.008 (3)	−0.004 (3)
O16	0.069 (4)	0.077 (4)	0.056 (3)	−0.006 (3)	−0.006 (3)	−0.009 (3)
C75	0.076 (6)	0.094 (7)	0.104 (8)	−0.011 (5)	−0.020 (6)	0.038 (7)
C76	0.044 (5)	0.120 (9)	0.144 (11)	0.006 (5)	0.012 (6)	0.041 (8)
C77	0.088 (7)	0.056 (5)	0.097 (7)	0.016 (4)	−0.022 (6)	0.009 (5)
C78	0.072 (5)	0.057 (4)	0.050 (4)	0.005 (4)	−0.005 (4)	0.005 (4)

### Bis[(2.2.2-cryptand)potassium] 2,2,4,6-tetraphenyl-1,3,5,2λ4,4,6-trioxatriborinan-2-ide cyanomethyldiphenylborate tetrahydrofuran disolvate (4) Geometric parameters (Å, º)

**Table:**

B1—O1	1.499 (8)	O8—C53	1.433 (7)
B1—O3	1.508 (8)	O9—C54	1.420 (7)
B1—C1	1.634 (10)	O9—C55	1.432 (7)
B1—C7	1.655 (10)	N2—C45	1.457 (9)
B2—O1	1.331 (8)	N2—C51	1.463 (8)
B2—O2	1.374 (8)	N2—C39	1.475 (9)
B2—C13	1.589 (9)	N3—C56	1.464 (8)
B3—O3	1.342 (8)	N3—C50	1.469 (8)
B3—O2	1.380 (8)	N3—C44	1.472 (9)
B3—C19	1.601 (9)	C39—C40	1.481 (12)
C1—C6	1.374 (9)	C41—C42	1.466 (12)
C1—C2	1.394 (9)	C43—C44	1.513 (10)
C2—C3	1.375 (9)	C45—C46	1.507 (9)
C3—C4	1.368 (11)	C47—C48	1.497 (9)
C4—C5	1.405 (12)	C49—C50	1.511 (10)
C5—C6	1.370 (11)	C51—C52	1.499 (10)
C7—C8	1.375 (10)	C53—C54	1.500 (9)
C7—C12	1.401 (9)	C55—C56	1.522 (9)
C8—C9	1.364 (12)	K2—O11	2.802 (4)
C9—C10	1.366 (14)	K2—O13	2.824 (4)
C10—C11	1.371 (13)	K2—O14	2.826 (4)
C11—C12	1.408 (11)	K2—O15	2.828 (4)
C13—C14	1.368 (10)	K2—O10	2.848 (5)
C13—C18	1.413 (9)	K2—O12	2.855 (5)
C14—C15	1.382 (10)	K2—N5	2.947 (5)
C15—C16	1.372 (12)	K2—N4	2.980 (7)
C16—C17	1.414 (13)	O10—C59	1.387 (10)
C17—C18	1.373 (10)	O10—C58	1.407 (12)
C19—C24	1.367 (9)	O11—C61	1.436 (10)
C19—C20	1.387 (9)	O11—C60	1.475 (9)
C20—C21	1.379 (9)	O12—C64	1.429 (11)
C21—C22	1.435 (11)	O12—C65	1.433 (10)
C22—C23	1.371 (10)	O13—C66	1.412 (8)
C23—C24	1.394 (9)	O13—C67	1.421 (9)
B4—C26	1.488 (10)	O14—C70	1.406 (9)
B4—C25	1.614 (10)	O14—C71	1.430 (7)
B4—C27	1.633 (11)	O15—C73	1.421 (7)
B4—C33	1.648 (11)	O15—C72	1.438 (7)
N1—C25	1.163 (10)	N4—C63	1.459 (11)
C27—C32	1.384 (10)	N4—C69	1.462 (10)
C27—C28	1.399 (11)	N4—C57	1.474 (12)
C28—C29	1.423 (13)	N5—C62	1.471 (9)
C29—C30	1.345 (14)	N5—C68	1.483 (9)
C30—C31	1.336 (14)	N5—C74	1.490 (8)
C31—C32	1.365 (11)	C57—C58	1.482 (16)
C33—C34	1.380 (9)	C59—C60	1.452 (12)
C33—C38	1.394 (11)	C61—C62	1.493 (11)
C34—C35	1.369 (10)	C63—C64	1.464 (14)
C35—C36	1.339 (10)	C65—C66	1.486 (12)
C36—C37	1.365 (10)	C67—C68	1.506 (10)
C37—C38	1.400 (12)	C69—C70	1.504 (10)
K1—O9	2.805 (4)	C71—C72	1.475 (9)
K1—O5	2.815 (4)	C73—C74	1.510 (11)
K1—O6	2.819 (4)	O16—C75	1.393 (12)
K1—O4	2.830 (5)	O16—C78	1.435 (9)
K1—O7	2.872 (4)	C75—C76	1.467 (14)
K1—O8	2.873 (4)	C76—C77	1.546 (17)
K1—N2	2.965 (5)	C77—C78	1.478 (13)
K1—N3	2.974 (5)	O17—C79	1.49 (3)
O4—C41	1.405 (9)	O17—C82	1.52 (3)
O4—C40	1.440 (11)	C79—C80	1.65 (3)
O5—C43	1.435 (8)	C80—C81	1.59 (4)
O5—C42	1.468 (8)	C81—C82	1.44 (4)
O6—C47	1.420 (7)	O17B—C79B	1.54 (3)
O6—C46	1.431 (7)	O17B—C82B	1.66 (3)
O7—C48	1.425 (7)	C79B—C80B	1.41 (3)
O7—C49	1.431 (8)	C80B—C81B	1.39 (3)
O8—C52	1.420 (7)	C81B—C82B	1.63 (3)
			
O1—B1—O3	109.9 (5)	C45—N2—K1	108.8 (3)
O1—B1—C1	110.1 (5)	C51—N2—K1	109.0 (3)
O3—B1—C1	107.2 (5)	C39—N2—K1	110.6 (4)
O1—B1—C7	108.8 (5)	C56—N3—C50	110.1 (5)
O3—B1—C7	109.8 (5)	C56—N3—C44	109.6 (5)
C1—B1—C7	111.0 (5)	C50—N3—C44	109.8 (5)
O1—B2—O2	122.2 (6)	C56—N3—K1	108.7 (3)
O1—B2—C13	120.2 (6)	C50—N3—K1	110.0 (3)
O2—B2—C13	117.6 (5)	C44—N3—K1	108.7 (4)
O3—B3—O2	123.0 (6)	N2—C39—C40	114.6 (6)
O3—B3—C19	120.5 (5)	O4—C40—C39	109.0 (6)
O2—B3—C19	116.5 (5)	O4—C41—C42	108.7 (6)
B2—O1—B1	123.3 (5)	C41—C42—O5	110.3 (5)
B2—O2—B3	118.5 (5)	O5—C43—C44	108.3 (5)
B3—O3—B1	121.9 (5)	N3—C44—C43	113.9 (5)
C6—C1—C2	116.4 (6)	N2—C45—C46	114.2 (5)
C6—C1—B1	119.4 (6)	O6—C46—C45	109.0 (5)
C2—C1—B1	124.1 (6)	O6—C47—C48	109.3 (5)
C3—C2—C1	123.1 (6)	O7—C48—C47	109.6 (5)
C4—C3—C2	119.0 (6)	O7—C49—C50	109.1 (6)
C3—C4—C5	119.7 (7)	N3—C50—C49	114.6 (5)
C6—C5—C4	119.6 (7)	N2—C51—C52	114.9 (5)
C5—C6—C1	122.3 (7)	O8—C52—C51	109.7 (6)
C8—C7—C12	118.1 (7)	O8—C53—C54	109.1 (5)
C8—C7—B1	123.0 (6)	O9—C54—C53	109.3 (5)
C12—C7—B1	118.9 (6)	O9—C55—C56	108.6 (5)
C9—C8—C7	121.1 (8)	N3—C56—C55	115.0 (5)
C8—C9—C10	121.5 (8)	O11—K2—O13	95.89 (15)
C9—C10—C11	119.7 (7)	O11—K2—O14	139.54 (15)
C10—C11—C12	119.4 (7)	O13—K2—O14	121.34 (15)
C7—C12—C11	120.3 (7)	O11—K2—O15	100.52 (14)
C14—C13—C18	116.2 (6)	O13—K2—O15	101.51 (13)
C14—C13—B2	122.1 (6)	O14—K2—O15	59.70 (12)
C18—C13—B2	121.7 (6)	O11—K2—O10	59.95 (16)
C13—C14—C15	120.1 (7)	O13—K2—O10	138.17 (15)
C16—C15—C14	123.1 (7)	O14—K2—O10	94.97 (15)
C15—C16—C17	118.9 (7)	O15—K2—O10	115.46 (16)
C18—C17—C16	116.3 (7)	O11—K2—O12	112.31 (15)
C17—C18—C13	125.3 (7)	O13—K2—O12	59.27 (15)
C24—C19—C20	118.6 (6)	O14—K2—O12	100.96 (14)
C24—C19—B3	121.8 (6)	O15—K2—O12	142.74 (15)
C20—C19—B3	119.6 (6)	O10—K2—O12	96.54 (17)
C21—C20—C19	121.7 (6)	O11—K2—N5	61.21 (15)
C20—C21—C22	120.0 (7)	O13—K2—N5	61.52 (15)
C23—C22—C21	116.5 (6)	O14—K2—N5	120.95 (13)
C22—C23—C24	122.7 (6)	O15—K2—N5	62.25 (13)
C19—C24—C23	120.4 (7)	O10—K2—N5	119.07 (17)
C26—B4—C25	110.9 (6)	O12—K2—N5	119.12 (15)
C26—B4—C27	113.2 (7)	O11—K2—N4	118.08 (18)
C25—B4—C27	104.2 (6)	O13—K2—N4	118.20 (18)
C26—B4—C33	109.1 (6)	O14—K2—N4	60.00 (14)
C25—B4—C33	106.6 (7)	O15—K2—N4	118.67 (14)
C27—B4—C33	112.5 (6)	O10—K2—N4	60.4 (2)
N1—C25—B4	171.6 (9)	O12—K2—N4	60.39 (17)
C32—C27—C28	115.2 (7)	N5—K2—N4	179.04 (16)
C32—C27—B4	125.9 (7)	C59—O10—C58	107.1 (7)
C28—C27—B4	118.8 (7)	C59—O10—K2	113.4 (4)
C27—C28—C29	120.6 (7)	C58—O10—K2	117.1 (5)
C30—C29—C28	119.6 (8)	C61—O11—C60	110.4 (6)
C31—C30—C29	120.7 (8)	C61—O11—K2	117.1 (4)
C30—C31—C32	120.4 (9)	C60—O11—K2	113.7 (4)
C31—C32—C27	123.3 (8)	C64—O12—C65	110.9 (7)
C34—C33—C38	115.1 (6)	C64—O12—K2	115.9 (4)
C34—C33—B4	120.8 (6)	C65—O12—K2	115.5 (4)
C38—C33—B4	124.2 (6)	C66—O13—C67	111.1 (5)
C35—C34—C33	122.8 (6)	C66—O13—K2	116.6 (4)
C36—C35—C34	121.7 (6)	C67—O13—K2	117.9 (4)
C35—C36—C37	118.3 (7)	C70—O14—C71	112.9 (5)
C36—C37—C38	120.8 (7)	C70—O14—K2	119.3 (4)
C33—C38—C37	121.1 (7)	C71—O14—K2	116.3 (3)
O9—K1—O5	97.61 (12)	C73—O15—C72	112.0 (5)
O9—K1—O6	120.79 (13)	C73—O15—K2	115.3 (3)
O5—K1—O6	136.77 (13)	C72—O15—K2	115.1 (3)
O9—K1—O4	138.46 (13)	C63—N4—C69	109.8 (7)
O5—K1—O4	59.89 (14)	C63—N4—C57	109.4 (7)
O6—K1—O4	95.88 (13)	C69—N4—C57	109.2 (7)
O9—K1—O7	100.40 (12)	C63—N4—K2	109.8 (4)
O5—K1—O7	97.57 (13)	C69—N4—K2	109.7 (4)
O6—K1—O7	59.09 (12)	C57—N4—K2	109.0 (6)
O4—K1—O7	115.96 (14)	C62—N5—C68	111.7 (5)
O9—K1—O8	59.14 (11)	C62—N5—C74	109.2 (6)
O5—K1—O8	116.81 (13)	C68—N5—C74	109.6 (5)
O6—K1—O8	100.66 (11)	C62—N5—K2	110.4 (4)
O4—K1—O8	97.99 (15)	C68—N5—K2	107.7 (4)
O7—K1—O8	140.91 (13)	C74—N5—K2	108.1 (3)
O9—K1—N2	119.17 (13)	N4—C57—C58	113.6 (7)
O5—K1—N2	118.79 (15)	O10—C58—C57	110.6 (9)
O6—K1—N2	60.90 (13)	O10—C59—C60	108.5 (6)
O4—K1—N2	60.26 (16)	C59—C60—O11	109.5 (6)
O7—K1—N2	118.90 (13)	O11—C61—C62	109.9 (6)
O8—K1—N2	61.17 (12)	N5—C62—C61	114.4 (5)
O9—K1—N3	61.21 (12)	N4—C63—C64	114.2 (6)
O5—K1—N3	61.12 (15)	O12—C64—C63	111.1 (8)
O6—K1—N3	118.86 (13)	O12—C65—C66	109.1 (6)
O4—K1—N3	119.57 (16)	O13—C66—C65	110.6 (6)
O7—K1—N3	60.81 (13)	O13—C67—C68	109.4 (5)
O8—K1—N3	119.23 (12)	N5—C68—C67	114.1 (5)
N2—K1—N3	179.61 (15)	N4—C69—C70	112.5 (7)
C41—O4—C40	108.8 (6)	O14—C70—C69	110.9 (6)
C41—O4—K1	116.5 (4)	O14—C71—C72	109.8 (5)
C40—O4—K1	118.1 (4)	O15—C72—C71	109.9 (5)
C43—O5—C42	110.1 (5)	O15—C73—C74	110.1 (5)
C43—O5—K1	117.3 (4)	N5—C74—C73	114.0 (6)
C42—O5—K1	113.6 (4)	C75—O16—C78	105.8 (7)
C47—O6—C46	110.5 (4)	O16—C75—C76	109.3 (9)
C47—O6—K1	117.6 (3)	C75—C76—C77	104.4 (8)
C46—O6—K1	118.1 (3)	C78—C77—C76	102.9 (8)
C48—O7—C49	110.8 (5)	O16—C78—C77	106.3 (7)
C48—O7—K1	115.1 (3)	C79—O17—C82	107.3 (16)
C49—O7—K1	116.0 (3)	O17—C79—C80	101.2 (15)
C52—O8—C53	110.3 (5)	C81—C80—C79	93 (2)
C52—O8—K1	114.9 (3)	C82—C81—C80	106 (3)
C53—O8—K1	114.0 (3)	C81—C82—O17	106 (2)
C54—O9—C55	110.0 (4)	C79B—O17B—C82B	103.9 (17)
C54—O9—K1	118.8 (3)	C80B—C79B—O17B	102.6 (19)
C55—O9—K1	118.7 (3)	C81B—C80B—C79B	113 (2)
C45—N2—C51	110.0 (6)	C80B—C81B—C82B	110.4 (18)
C45—N2—C39	109.9 (5)	C81B—C82B—O17B	92.6 (15)
C51—N2—C39	108.5 (5)		
